# Generally-healthy individuals with aberrant bowel movement frequencies show enrichment for microbially-derived blood metabolites associated with impaired kidney function

**DOI:** 10.1101/2023.03.04.531100

**Published:** 2023-03-06

**Authors:** James P. Johnson, Christian Diener, Anne E. Levine, Tomasz Wilmanski, David L. Suskind, Alexandra Ralevski, Jennifer Hadlock, Andrew T. Magis, Leroy Hood, Noa Rappaport, Sean M. Gibbons

**Affiliations:** 1 Institute for Systems Biology, Seattle, WA 98109, USA; 2 Department of Bioengineering, University of Washington, Seattle, WA 98195, USA; 3 Seattle Children’s Hospital, Seattle, WA 98105, USA; 4 Phenome Health, Seattle, WA 98109; 5 Department of Immunology, University of Washington, Seattle, WA 98195, USA; 6 Paul G. Allen School of Computer Science & Engineering, University of Washington, Seattle, WA 98195, USA; 7 Department of Genome Sciences, University of Washington, Seattle, WA 98195, USA; 8 eScience Institute, University of Washington, Seattle, WA 98195, USA

## Abstract

**Objective:**

Bowel movement frequency (BMF) variation has been linked to changes in the composition of the human gut microbiome and to many chronic conditions, like metabolic disorders, neurodegenerative diseases, chronic kidney disease (CKD), and other intestinal pathologies like irritable bowel syndrome (IBS) and inflammatory bowel disease (IBD). Slow intestinal transit times (constipation) are thought to lead to compromised intestinal barrier integrity and a switch from saccharolytic to proteolytic fermentation within the microbiota, giving rise to microbially-derived toxins that may make their way into circulation and cause damage to organ systems. However, these phenomena have not been characterized in generally-healthy populations, and the connections between microbial metabolism and the early-stage development and progression of chronic disease remain underexplored.

**Design:**

Here, we examine the phenotypic impact of BMF variation across a cohort of over 2,000 generally-healthy, community dwelling adults with detailed clinical, lifestyle, and multi-omic data.

**Results:**

We show significant differences in key blood plasma metabolites, proteins, chemistries, gut bacterial genera, and lifestyle factors across BMF groups that have been linked, in particular, to inflammation and CKD severity and progression.

**Discussion:**

In addition to dissecting BMF-related heterogeneity in blood metabolites, proteins, and the gut microbiome, we identify self-reported diet, lifestyle, and psychological factors associated with BMF variation, which suggest several potential strategies for mitigating constipation and diarrhea. Overall, this work highlights the potential for managing BMF to prevent disease.

## INTRODUCTION

The gut microbiome influences human health in a number of ways, from mediating early life immune system development [1,2], to determining personalized responses to nutritional interventions [3,4] and influencing the central nervous system [5,6]. Stool transit time, defined as the rate at which stool moves through the gastrointestinal tract, is a major determinant of the composition of the human gut microbiota [7]. Transit time is affected by diet, hydration, physical activity, host mucus production, microbe- and host-derived small molecules (e.g., bile acids or neurotransmitters), and peristaltic smooth muscle contractions in the gastrointestinal tract [8,9]. Stool transit time can be inferred or measured using the Bristol Stool Scale [10], edible dyes [7], indigestible food components (e.g., corn) [11], or self-reported bowel movement frequency (BMF) [12]. Aberrant gastrointestinal transit times have been implicated as risk factors in a number of chronic diseases [13–15].

Shorter stool transit times (e.g. diarrhea, defined as more than three watery stools per day), have been associated with lower gut microbiome alpha diversity, increased susceptibility to enteric pathogens, and poorer overall health [12,16–18]. Longer stool transit times (e.g. constipation, defined as fewer than three hard, dry stools per week), have been associated with higher gut microbiome alpha diversity, with an enrichment in microbially-derived urinary metabolites known to be hepatotoxic or nephrotoxic, and with an increased risk for several chronic medical conditions, including neurological disorders and chronic kidney disease (CKD) severity [13,19–21]. Interestingly, the relationship between higher gut alpha-diversity and constipation contrasts with the common belief that increased diversity is a positive marker of gut health, and suggests a more complex relationship between gut commensal diversity and human health [12,13].

Constipation is a known risk factor for CKD severity and end-stage renal disease (ESRD) progression [22,23]. In one study, up to 71% of dialysis patients suffered from constipation [24], while the prevalence of constipation in the general population was 14.5% in adults under 60 years old and 33.5% in those over 60 [25]. A nationwide study of veterans found an incrementally higher risk for renal disease progression in those who reported increasingly severe constipation [26]. However, while it is clear that morbidity and mortality risk worsen with constipation in those with active CKD, potential connections between the gut microbiota and the development and early-stage kidney disease are not yet established. Both constipation and CKD associate with declines in gut microbiota-mediated short-chain fatty acid (SCFA) production and a rise in the production of amino acid putrefaction byproducts, including several toxic metabolites, such as p-cresol sulfate (PCS), which has been causally implicated in renal tissue damage [27]. This is consistent with a community-wide transition from saccharolytic to proteolytic fermentation due to the exhaustion of dietary fiber with longer GI transit times [13,28]. Thus, while the relationships between BMF in healthy individuals and future CKD pathogenesis, along with damage to other organ systems like the central nervous system, are not yet understood, the gut metabolic phenotype associated with low BMF in a prodromal cohort suggests an early causal connection.

In this study, we focus on categories of self-reported BMF in a large population of generally-healthy individuals with a wide range of molecular phenotypic data, including data on gut microbiome composition, in order to quantify the phenotypic impact of BMF on blood plasma metabolites, blood proteins, clinical chemistries, and gut microbiome composition in a pre-disease context. By exploring the molecular phenotypic consequences of BMF variation in a generally-healthy cohort, we hope to identify early-stage biomarkers for CKD risk and provide further insight into the possible causal connections between BMF and several chronic, non-communicable diseases. Finally, we assess how demographic, dietary, lifestyle, and psychological factors are associated with variation in BMF, in order to identify potential interventions for manipulating BMF and BMF-associated phenotypes.

## RESULTS

### A cohort of generally-healthy individuals

**Figure 1.**
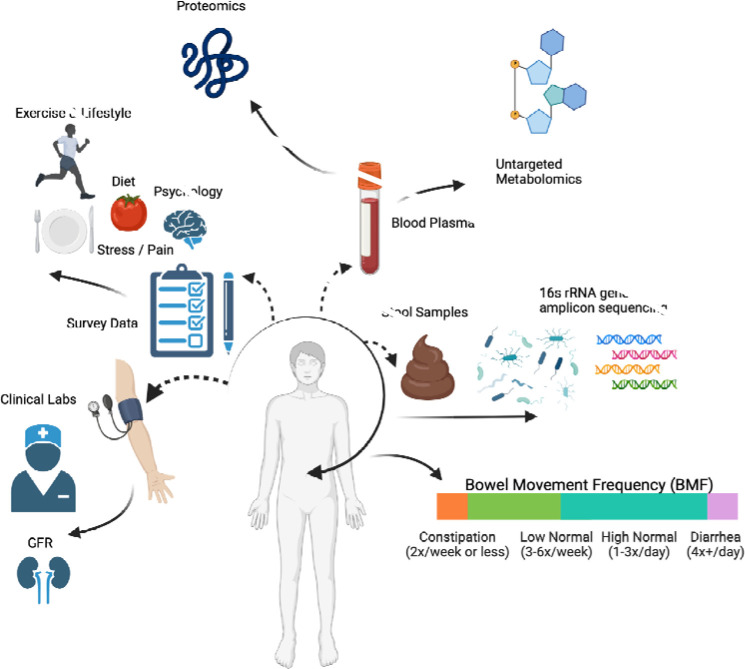
Data collection strategy. Arivale participants had their multi-omics, survey, and clinical data collected through various methods: interviewing, blood plasma collection, and stool samples. Interview data consisted of several questions with categorical responses, either ordinal or binary (True/False) answers (which were excluded in this analysis), which were then used in ordinal POLR to determine likelihoods of different response categories across BMF and its covariates. Clinical labs, untargeted metabolomics, and proteomics data were obtained from collected blood plasma samples (the earliest sample available per participant in the cohort). Gut microbiome ASV data were collected from stool samples provided using an at-home kit. BMF data were determined as categorical ranges of reported bowel movements per week or day depending on the response to lifestyle questionnaire data from the interviews.

**Figure 2.**
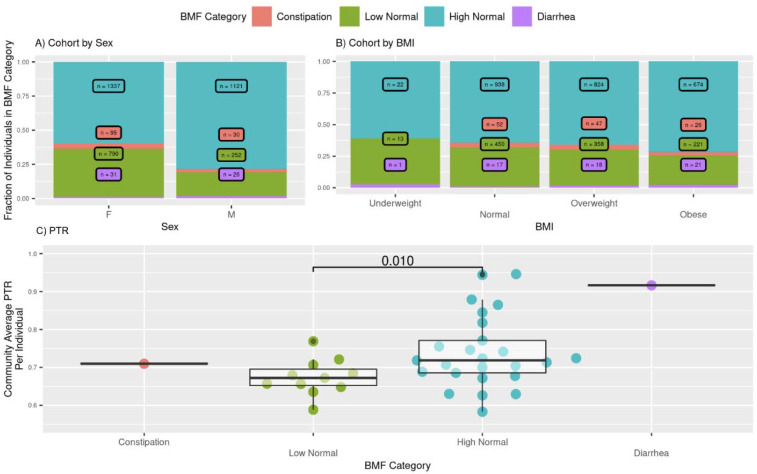
Distribution of BMF categories across sex and BMI groups and the relationship between average community growth rate and BMF. (A-B): Significant unevenness in the distribution of BMF across sex and BMI are highlighted here. POLR was used to regress BMF against the covariates sex, age, BMI, and eGFR. The result was that only sex (P = 1.23E-23) and BMI (P = 5.09E-6) were significantly associated with variations in BMF. (C): Community Average PTR Per Individual (the mean growth rate across all growth rates of all taxa for a given individual). There is a significant difference (linear regression, P value = 1.56E-2; post-hoc t-test P value = 1.0E-2) between the higher and lower BMF “normal” categories, showing a general directional trend of increasing community average PTR with rising BMF level, indicative of higher BMF representing a higher “flow rate” of material through the gut which is associated with higher growth community growth rates.

3,955 Arivale Scientific Wellness program participants with BMF data were analyzed (see Materials and Methods). Arivale, Inc. (USA), was a consumer scientific wellness company that operated from 2015 until 2019. Briefly, participants consented to having their health, diet, and lifestyle surveyed through an extensive questionnaire, along with blood and stool sampling for multi-omic and blood plasma chemistries data generation (Fig. 1). Of those participants that self-reported their ethnicity, 80.5% identified as “White”, 10.2% identified as “Asian”, 2.9% identified as “Black or African-American”, 0.3% identified as “American Indian or Alaska Native”, 0.8% identified as “Native Hawaiian or other Pacific Islander”, and 5.4% identified as “Other”. Additionally, Arivale participants responded 92.9% “Non-Hispanic” versus 7.1% “Hispanic”. Of the 109 Hispanic cohort participants, 59.6% also self-reported white. Respondents were in the United States, predominantly from the Pacific West. These individuals were generally-healthy, non-hospitalized and aged between 19 and 87 years old. The population was 61% female with a mean ± s.d. body mass index of 27.47 ± 6.15. Self-reported BMF values (responses to typical number of bowel movements per week) were grouped into four categories (Fig. 1), which we defined as: “constipation” (≤ 2 bowel movements per week), “low-normal” (3–6 bowel movements per week), “high-normal” (1–3 bowel movements per day), and “diarrhea” (4 or more bowel movements per day). We first looked at potential associations between BMF and relevant covariates: sex, age, BMI, and estimated glomerular filtration rate (eGFR), a measure of renal function (N = 3,682; Fig. 2; Table S2). When BMF was coded as an ordinal dependent variable and regressed using ordered proportional odds logistic regression (POLR), only BMI (POLR, FDR-corrected p = 5.09E-6) and sex (POLR, FDR-corrected P = 1.23E-23) showed significant, independent associations with BMF (Table S2), with females and individuals with lower BMIs tending to report lower BMFs (Fig. 2). All covariates listed above were included in downstream regressions, independent of whether or not they showed a direct association with BMF. The high-normal BMF group was chosen as the reference for all downstream regressions throughout the manuscript where BMF was encoded as a categorical variable.

### Gut microbiome composition and activity across BMF categories

For a small subset of the Arivale participants (N=38) shotgun metagenomic sequencing data were available in addition to 16S rRNA gene amplicon sequencing data. For this subset, we calculated peak-to-trough ratios (PTR, a proxy for growth/replication rate) for abundant bacterial taxa within each sample. We saw a significant positive pairwise association between community-average PTRs and BMF (Fig. 2C, post-hoc t-test low-normal vs. high-normal, P = 0.010), which suggests that we tend to capture a larger number of commensal bacteria in their exponential growth phase when we sample them from individuals with higher BMFs.

**Figure 3.**
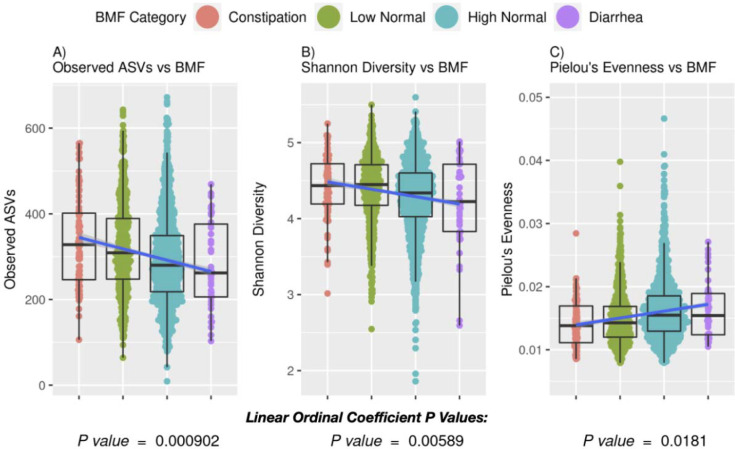
Associations between gut microbiome alpha-diversity measures and BMF. (A) The numbers of taxonomic groups per BMF category, representing the richness of the BMF cohort (ordinal BMF variable - ANOVA P value: 9.02E-4). (B) The number of different taxonomic groups (variety) per BMF category, representing the alpha diversity of the BMF cohort (ordinal BMF variable - ANOVA P value: 5.89E-3). (C) The distribution of abundances of the taxonomic groups, determined proportionally by dividing the diversity by the richness of the cohort (evenness, ordinal BMF variable - ANOVA P value: 1.81E-2). The evenness decreases with BMF, suggesting slow colonic transit times (constipation) correspond to having a higher ratio of richness to diversity and lower evenness.

Next, we looked at a larger cohort of individuals with 16S amplicon sequencing data from stool (N=2,709). Amplicon sequence variant (ASV) richness (linear regression, P = 9.02E-4) and Shannon diversity (linear regression, P = 5.89E-3) were both negatively associated with BMF, independent of covariates (BMF encoded as an ordinal variable with a linear coefficient, Fig. 3). Pielou’s evenness, on the other hand, was positively associated with BMF (linear regression, P = 1.81E-2), independent of covariates (Fig. 3). Thus, slow colonic transit times as seen in constipation correspond to a higher community richness and lower community evenness.

**Figure 4.**
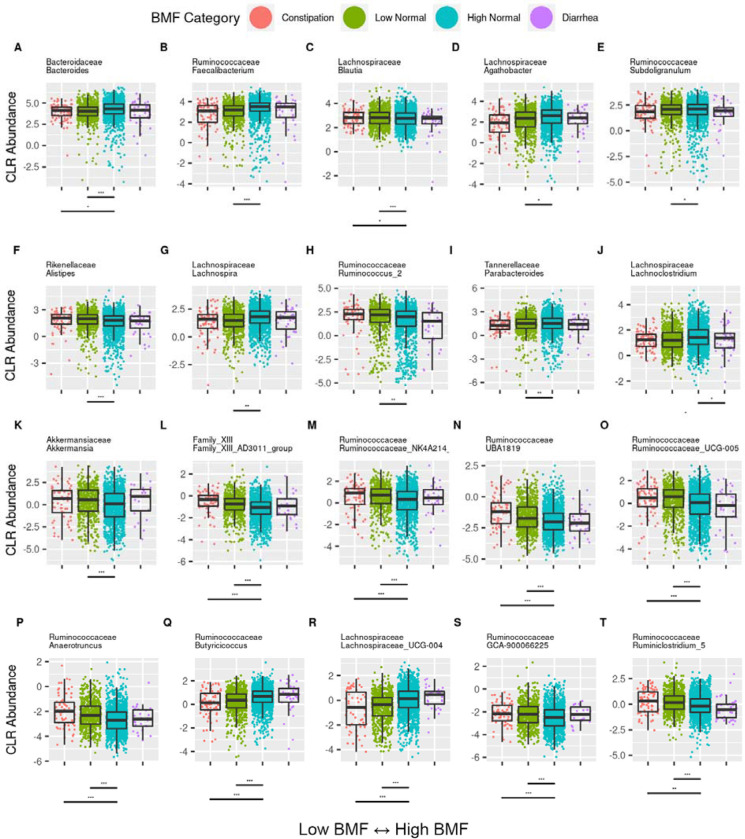
Significant BMF associations for the 10 most abundant genera, *Akkermansia*, and another 9 genera with the lowest remaining P values. The top 10 most abundant significant taxa from the fecal samples and ASV CORNCOB analysis (A-J), Akkermansia (K), and the top 9 most significant taxa not already included in the most abundant list (L-T). Lines beneath each plot denote significant differences from the reference category, and asterisks denote FDR-corrected significance threshold. (***): P < 0.0001, (**): 0.0001 < P < 0.01, (*): 0.01 < P < 0.05.

Differential abundance analysis of the commensal gut bacterial genera across BMF categories was conducted using beta-binomal regression (CORNCOB) with BMF encoded as a categorical variable. Of the 68 genera that passed our prevalence filter (i.e., detection across ≥ 30% of the individuals), 47 were significantly associated with BMF (see Table S3 for detailed list of coefficients and p-values), independent of covariates and following an FDR correction for multiple tests on the likelihood ratio test (LRT) P values (LRT, FDR-corrected P < 0.05). Of the 47 significant taxa, we plotted the top ten most abundant (Fig. 4A–J and Table S4) and the following top 10 most significant taxa (i.e., according to the LRT FDR-corrected P value), including *Akkermansia* (Fig. 4K–T). *Bacteroides, Blautia, Family_XIII_AD3011_group, Ruminococcaceae_NK4A214_group,* Ruminococcaceae *UBA1819, Ruminococcaceae_UCG-005, Anaerotruncus, Butyricicoccus, Lachnospiraceae_UCG-004*, *Ruminococcaceae GCA-900066225, Ruminococcaceae Ruminiclostridium_5* were each differentially abundant between constipation and the high-normal (reference) category (LRT, FDR-corrected ratio test P < 0.05). Agathobacter, Subdoligranulum, Lachnospira, Lachnoclostridium, Butyricicoccus, and Lachnospiraceae_UCG-004 all showed decreasing abundances with lower BMFs (LRT, FDR-corrected P < 0.05). *Lachnoclostridium* rose in abundance with BMF, and was highest in individuals who reported having diarrhea (LRT, FDR-corrected P < 0.05). In contrast, *Blautia, Alistipes, Ruminococcaceae UCG-005, Ruminococcus_2, UBA1819, Ruminococcaceae_NK4A214_group, Anaerotruncus, GCA-900066225*, and *Ruminiclostridium_5* showed the opposite behavior, where decreasing BMF was associated with a increasing abundance of these taxa (LRT, FDR-corrected P < 0.05). Some genera appeared to exhibit local minima or maxima (U-shaped vs. peaked relationship with BMF), indicating non-linear trends. These taxa included Bacteroides, Faecalibacterium, *GCA-900066225*, *Akkermansia*, and a genus from Family XIII AD3011. However, we had limited power to confidently identify putative non-monotonic trends due to the small number of individuals in the constipation and diarrhea groups.

### Variation in blood metabolites across BMF categories

**Figure 5.**
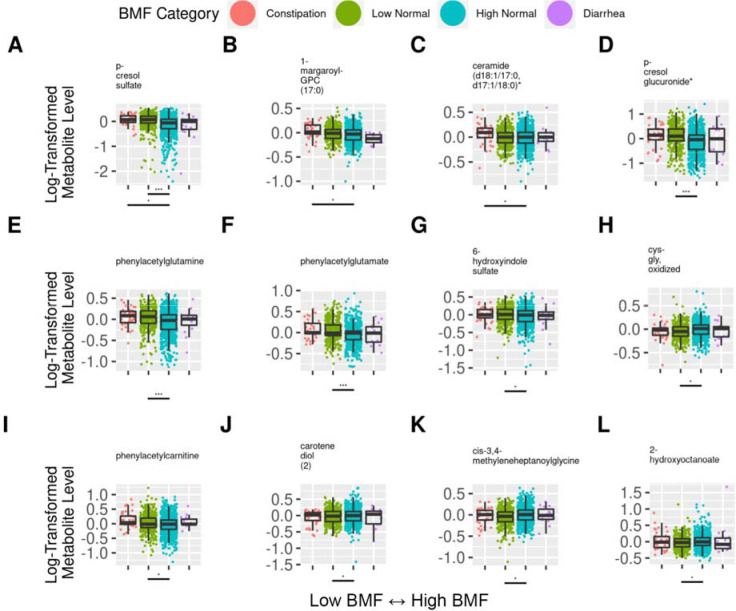
Top 12 BMF-associated blood plasma metabolites with annotations. (A-L) The 12 significant blood plasma metabolites from the LIMMA metabolomics analysis with available annotations. Lines beneath each plot denote significant differences from the reference category, and asterisks denote FDR-corrected significance threshold. (***): P < 0.0001, (**): 0.0001 < P < 0.01, (*): 0.01 < P < 0.05.

Blood metabolite-BMF regression analyses were run using a generalized linear modeling (GLM) framework (LIMMA), with BMF as a categorical variable. Of the metabolites that passed our abundance and prevalence filters (N=1,296, see Materials and Methods), 27 unique metabolites were significantly associated with BMF (0 with diarrhea, 24 with low-normal, 4 with constipation, and 1 overlapping metabolite, PCS, associated with both low-normal and constipation), independent of covariates and following an FDR correction for multiple tests (GLM, FDR-corrected P < 0.05, Fig. 5, Table S5). 20 out of 27 metabolites were enriched in the low-normal and/or constipation BMF groups, showing a monotonically decreasing trend with BMF, while the rest showed a monotonically increasing trend (Fig. 5). One metabolite, phenylacetylcarnitine, showed a slight, apparent local minimum (“U-shaped” behavior) with lowest levels in the high-normal BMF category (Fig. 5). Several unannotated metabolites (e.g. X-12544) showed significant associations with BMF (GLM, FDR-corrected P < 0.05), but their identities and physiological roles are unknown (Table S5).

### Blood plasma chemistries across BMF categories

**Figure 6.**
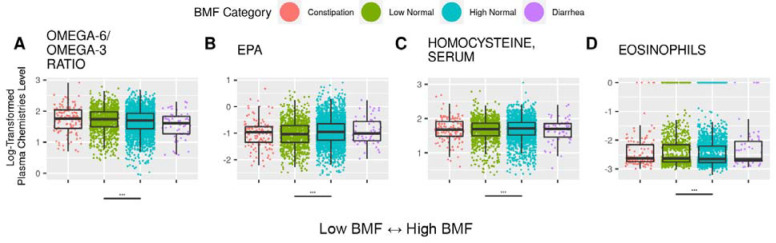
Blood plasma chemistries significantly associated with BMF. The 4 blood plasma chemistries features that showed significant associations with BMF. Lines beneath each plot denote significant differences from the reference category, and asterisks denote FDR-corrected significance threshold. (***): P < 0.0001, (**): 0.0001 < P < 0.01, (*): 0.01 < P < 0.05.

Of the 68 blood plasma chemistries tested, four were significantly different across BMF categories after adjusting for covariates and multiple-testing (N=3,682, GLM, FDR-corrected P < 0.05). These included Omega-6/Omega-3 ratio, eicosapentaenoic acid (EPA), homocysteine, and eosinophils levels in the blood (Fig. 6). All of these were elevated in the low-normal BMF category compared to the high-normal reference (FDR-corrected P < 0.05), except for EPA, which was lower in the low-normal BMF group (Fig. 6 and Table S6).

### Blood proteins across BMF categories

**Figure 7.**
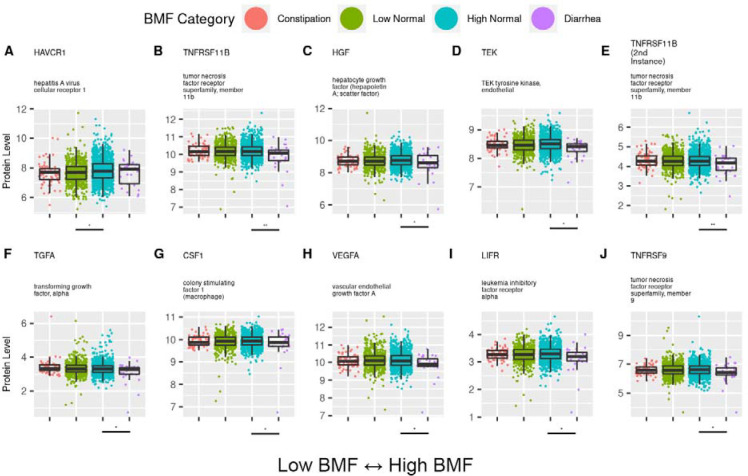
Selected blood plasma proteins significantly associated with BMF. Top 10 most significant blood plasma protein results with associated genes and annotated descriptions from the LIMMA proteomics analysis (A-J). Lines beneath each plot denote significant differences from the reference category, and asterisks denote FDR-corrected significance threshold. (***): P < 0.0001, (**): 0.0001 < P < 0.01, (*): 0.01 < P < 0.05.

Of the 274 blood proteins that passed our prevalence filter (see Materials and Methods), 26 showed significant associations with BMF after adjusting for covariates and multiple-testing (N=1,999, GLM, FDR-corrected P < 0.05). Hepatitis A virus cellular receptor 1 (HAVCR1) was depleted in the low-normal BMF category, relative to the reference group (GLM, FDR-corrected P < 0.05). The remaining 25 proteins were significantly depleted in the high BMF (diarrhea) group, relative to the reference group (GLM, FDR-corrected P < 0.05). The most significant diarrhea-related protein (GLM, FDR-corrected P < 0.05) was TNFRSF11B (tumor necrosis factor receptor superfamily, member 11b; Fig. 7, Table S7).

### Self-reported diet, lifestyle, anxiety and depression histories associated with BMF categories and demographic covariates

**Figure 8.**
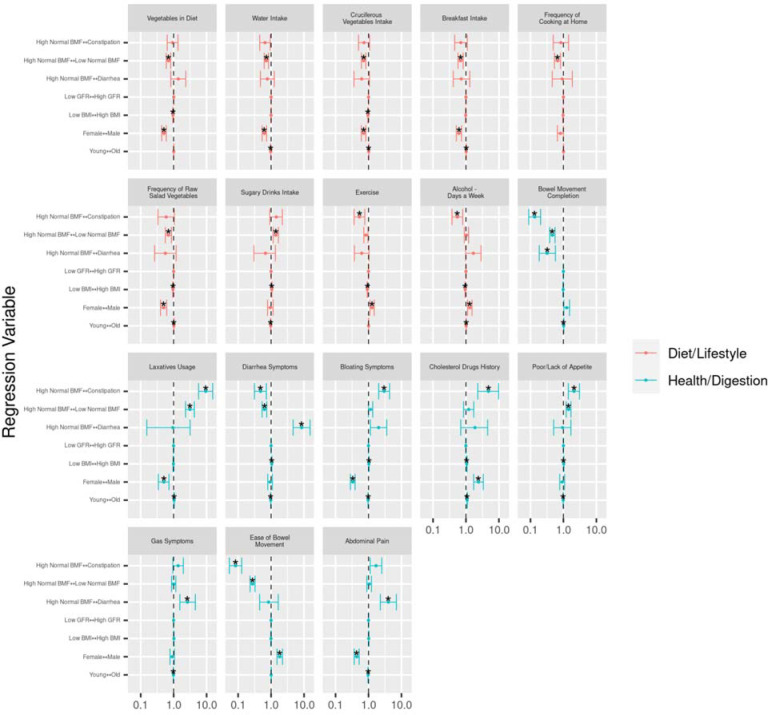
Ordinal regression odds ratio for health, diet, and lifestyle survey data vs BMF and covariates. Response variables are colored by category: questions related to diet, exercise, and lifestyle (Diet/Lifestyle), questions related to current digestive symptoms/function, health/medication history, and appetite (Health/Digestion), and questions related to the Big 5 Personality Test, mood/behavior or pain (Psychological). The BMF reference category was “high-normal” BMF (7–21 bowel movements per week). Each tick on the vertical axes represents a directional association in likelihood across the horizontal axis. The center line over the plots at x = 1.0 represents an equal likelihood of reporting an increase in number, intensity, frequency, or agreement (depending on the response variable) between the left side of the arrow on the vertical axis tick and the right side of the arrow on the vertical axis tick. A confidence interval that does not span the center line is significantly associated with the independent variable on the vertical axis tick. (*): FDR-corrected P-value < 0.05.

182 survey questions on mental and physical health, diet, and lifestyle were examined from 3,002 participants from the Arivale cohort in order to identify covariate-independent associations with BMF. Tests were run using the “polr” package in R (ordinal regression)[29], including the same set of covariates from the prior analyses, and with BMF coded as a categorical variable with high-normal BMF as the reference group (Fig. 8).

Response categories for each question ascended ordinally in value or intensity (i.e., low to high), so that a positive association represented an increase in that variable. Across the 182 questions, the top results with significant odds ratios related to BMF categories were displayed relative to high-normal BMF (Fig. 8), colored by the variable category (Diet/Lifestyle or Digestion/Health). BMI, age, and sex were also associated with many of these questionnaire-derived features, independent of BMF (Fig. 8). In particular, females took more laxatives, ate more vegetables (including salad and cruciferous vegetables), drank more water, ate breakfast more often, and suffered from greater abdominal pain and bloating. Males, on the other hand, tended to exercise more frequently, drank alcohol more frequently, had an easier time passing bowel movements, and were more likely to have used cholesterol-reducing drugs (Fig. 8). Constipation was negatively associated with exercise, alcohol intake, bowel movement completion, diarrhea symptoms, and ease of bowel movement, and positively associated with bloating, cholesterol drug use, reduced appetite, and reported laxative usage, independent of covariates (Fig. 8). Membership in the diarrhea BMF category was positively associated with self-reported diarrhea (i.e., a separate question from BMF on the questionnaire), increased bloating, and abdominal pain (Fig. 8).

A subset of participants self-reported their history of depression and anxiety, including: “self-current”, “self-past”, and “family” history of depression and anxiety (see Supplement). After logistic regression, one question related to “self-current” history of depression appeared marginally significant (logistic regression, FDR-corrected P < 0.1), with a “true” response associated with constipation. Similarly, questions related to a “self-past” (any time) history of anxiety (logistic regression, FDR-corrected P = 0.01) and a more recent “self-past” (within the last year) history of anxiety (logistic regression, FDR-corrected P = 0.048) were significantly associated with constipation.

## DISCUSSION

In this study, we delve into the multi-omic fingerprint of cross-sectional BMF variation in a large, generally-healthy population. We find that aberrant BMFs are associated with a wide array of phenotypic features, from changes in the ecological composition of the gut microbiota, to variation in plasma metabolites, clinical chemistries, and blood proteins. Overall, we observe an enrichment of microbially-derived uremic toxins resulting from protein fermentation in individuals with lower BMFs. These toxins have been implicated in disease progression and mortality in CKD [23,30] and many of the same metabolites have been associated with other chronic diseases like neurodegeneration [31,32]. We suggest that BMF should be managed throughout the lifespan in order to minimize the build-up of microbially-derived toxins in the blood and to prevent chronic disease. We provide a number of common-sense dietary and lifestyle suggestions for managing BMF, which emerge from our analysis of this generally-healthy cohort.

### Diet, lifestyle, mood, and demographic factors associated with BMF variation

Of the core set of covariates used in these analyses, only sex and BMI were independently associated with BMF, with females and individuals with lower BMIs showing lower average BMF (Fig. 2). Prior work has shown that women are at higher risk of kidney dysfunction [33] and that both BMF and kidney function decline with age [34,35]. In addition to demographic factors associated with BMF, the questionnaire results indicate a number of dietary and lifestyle factors that influence BMF, like exercise frequency, eating fruits and vegetables (i.e., sources of dietary fiber), sleep, and stress (Fig. 8). We also saw evidence that constipation was marginally associated with depression and significantly associated with anxiety, which aligns with prior work showing higher prevalence of anxiety and depression (between 22–33%) on the Hospital Anxiety and Depression Scale (HADS) and the Mini International Neuropsychiatric Interview (MINI) in patients with chronic constipation [36]. The strong positive association between reported cholesterol drug use and constipation suggests that these drugs may influence BMF directly, or perhaps that a “heart healthy” diet/lifestyle that precludes the need for cholesterol medication promotes a healthier BMF range. Diets enriched in complex plant-based carbohydrates, such as starches and fibers, encourage saccharolytic fermentation in the gut microbiome, which likely reduces the level of proteolytic fermentation associated with kidney disease risk and other GI symptoms (Fig. 8).

### The association between BMF and chronic disease may be mediated by the gut microbiota

The barrier integrity of the intestinal epithelium, as well as gastrointestinal peristalsis, can be impaired by the enrichment or depletion of certain microbially-derived metabolites [28,37]. BMF-related changes in the composition of the gut microbiota observed in this study reveal a reduction in SCFA-producing genera, like *Bacteroides* and *Faecalibacterium*, in the aberrant BMF groups. Reduced SCFA production is known to weaken smooth muscle contractions that drive peristalsis [38–40], acting as a positive feedback on constipation, and inducing mechanical damage to the epithelium [41–43], which may contribute to subclinical inflammation and disruption of epithelial integrity [30,44,45]. This subclinical inflammation and epithelial damage may give rise to chronic peripheral and systemic inflammation over time and allow for excess luminal metabolites to leak into the blood, which can drive tissue damage throughout the body and exacerbate conditions like CKD [30,46–48].

Many of the genera and metabolites that were associated with constipation in this study have been associated with constipation in other disease cohorts and with a variety of risk factors for chronic diseases, like CKD, cardiovascular disease, and metabolic syndrome [8,23,49,50]. *Alistipes* and *Ruminococcus* were enriched in end-stage renal disease (ESRD) patients [51], as well as in our generally-healthy cohort at lower BMF levels (Fig. 4). In general, families like Ruminococcaceae and Lachnospiraceae dominate the pool of significant BMF-microbiome hits (Fig. 4). In particular, *Roseburia*, a genus in the Lachnospiraceae family observed to be lower in abundance at all stages of CKD and ESRD [52], was found to be lower in abundance in individuals with lower BMFs in our cohort (Fig. 4). Akkermansia, a mucus-degrading genus generally associated with metabolic health [53], but also enriched in patients with Parkinson’s disease (PD) and in constipated individuals [32,54], was enriched at lower BMF in our cohort (Fig. 4). Akkermansia was positively associated with constipation across several studies [54], likely due to its specialization on breaking down host mucus rather than dietary substrates, but its absence also appears to have a detrimental impact on metabolic health and CKD progression [53,55,56]. Finally, we saw that the average gut bacterial community replication rate was positively associated with BMF (Fig. 2) and negatively associated with the production of several protein fermentation byproducts that are known uremic toxins (Fig. 5). Findings such as these suggest that constipation may drive pre-clinical risk and progression towards chronic diseases, mediated in part by BMF-induced switch from saccharolytic to proteolytic metabolism in the gut microbiota.

### BMF-associated blood metabolites are implicated in chronic disease risk and severity

Several blood metabolites found to be enriched at lower BMF were gut microbiome-derived uremic toxins linked to kidney function decline and neurodegenerative diseases. PCS, for example, has been associated with deteriorating kidney function and with damage to nephrons [57,58]. PCS showed the strongest association with BMF (Fig. 5), exhibiting a dose-response effect, increasing substantially in both the low-normal and constipation categories (Fig. 5). P-cresol glucuronide (PCG) is another uremic toxin, derived from microbe-produced p-cresol, which was significantly enriched at lower BMF (Fig. 5). Overall, we see an enrichment in several microbially-derived toxins in the blood of generally-healthy individuals with lower BMFs, like PCS, PCG, phenylacetylglutamine, 6-hydroxyindole sulfate, and phenylacetylcarnitine [58–60], which may drive long-term chronic disease risk.

### BMF-associated blood plasma chemistries results linked to inflammation and diet

Eicosapentaenoic acid (EPA) levels were lower in the lower-BMF groups (Fig. 6). Higher levels of EPA have been associated with lower inflammation [61] and lower cardiovascular disease risk [62]. Conversely, the Omega-6/Omega-3 ratio, homocysteine levels, and eosinophil counts, have all been positively associated with inflammation [63,64], and these features were elevated in the low-normal BMF group (Fig. 6). The Omega-6/Omega-3 ratio, in particular, may be related to higher levels of pro-inflammatory Omega-6 lipids and lower levels of anti-inflammatory Omega-3 lipids in the diet [65]. A diet enriched in processed foods and animal products is known to drive increased risk of chronic kidney disease [66,67]. The directionality of these associations point towards lower BMFs being associated with higher systemic inflammation, which may lead to increased chronic disease risk potentially through compromised gut epithelia.

### BMF-associated proteins connected to inflammation and renal injury

Hepatitis A virus cellular receptor 1 (HAVCR1) was the only protein that was depleted in the low-normal BMF group (Fig. 7A). HAVCR1 is, notably, an early biomarker for acute renal injury and a predictor of long-term renal disease, as it is shed into the urine following kidney injury [68]. Tumor necrosis factor receptor superfamily, member 11b (TNFRSF11B) showed the strongest association with BMF and was enriched in individuals with diarrhea (Fig. 7B). TNFRSF11B dysregulation has been associated with osteoporosis and with a number of cancers, and TNFSF members are involved in the pathogenesis of irritable bowel syndrome (IBS), a disease often associated with diarrhea [69,70]. The remaining proteins associated with aberrant BMFs were related to inflammation and undesirable immune responses, organ damage, and cancer (Fig. 7) [71,72].

### Current limitations and considerations on designing future research

There are some important limitations to consider when interpreting the results of this study. The generally-healthy cohort studied here was overwhelmingly “White”, predominantly female, and from the West Coast of the US, which limits the generalizability of these results. In addition, the diet, lifestyle, and mood data were self-reported and subject to biases and errors, and are not indicative of clinical diagnoses. In designing future follow-up trials, it would be ideal to manage BMF as a preventative measure for chronic disease and to target interventions that are low-risk with fewer side effects than drugs like laxatives. For example, BMF can be managed through exercise, hydration, and diet. However, high-fiber diets can lead to bloating and other issues in those with active disease. CKD patients, usually on multiple medications that may affect gut health and BMF, often need to eat a diet that restricts many plant-based fiber-rich foods because they contain high levels of potassium and phosphorus [73]. However, these low-fiber diets may act as a positive feedback on constipation and inflammation, as they promote protein fermentation in the gut. This highlights the importance of intervening at the prodromal stage, before disease manifests, when a healthy, plant-based diet is well-tolerated by the individual. Alternatively, low-potassium and low-phosphorus, high-fiber diets could be formulated for CKD patients. Ultimately, future work should be done to assess the potential for managing BMF throughout the lifespan to reduce chronic disease risk.

## Conclusion

Bowel movement abnormalities, such as constipation or diarrhea, have been linked to diseases ranging from enteric infections [18], CKD, and IBD to dementia-related neurodegenerative diseases like Alzheimer’s disease (AD) and PD [31,74,75]. Indeed, we see many of the phenotypic markers of these diseases manifested in generally-healthy individuals who report having aberrant BMFs, with constipation in particular associated with a build up of microbially-derived uremic toxins in the blood. Mitigating chronic constipation may be key to reducing uremic, hepatic, and neurological toxin build-up in the blood. Our results underscore common-sense dietary and lifestyle changes, like increasing dietary fiber intake, eating a lower protein diet and exercising more, may help to normalize BMF and reduce BMF-associated phenotypic risk factors for chronic disease, well before the onset of disease.

## MATERIALS AND METHODS

### Institutional review board approval for the study

The procedures for this study were reviewed and approved by the Western Institutional Review Board, under the institutional review board study number 20170658 for the Institute for Systems Biology and 1178906 for Arivale, Inc.

### Patient and Public Involvement Statement

There was no patient or public involvement in the conception or implementation of this research study.

### Generally-healthy cohort

All study participants were subscribers in the Arivale Scientific Wellness program (2015–2019) and provide informed consent for the use of their anonymized, deidentified data for research purposes. Participants were community-dwelling, representative of the populations in Washington State and California (which are slightly leaner and healthier than other parts of the USA), over the age of 18, non-pregnant, but were not screened for the presence or absence of any particular disease. Participants provided questionnaire data, along with blood and stool samples that were used to generate blood plasma metabolomics, proteomics, chemistries, and gut microbiome data (Fig 1 and Table S1).

Only baseline time point samples were used for each participant, prior to the beginning of a personalized wellness intervention. A 70% prevalence filter was implemented across the gut microbiome, blood plasma metabolomics, proteomics, chemistries, and ordinal questionnaire data analyses. This meant that each final feature in the data could contain no more than 30% missing data from the final cohort of samples in order to be retained for downstream analysis. For microbiome analyses, a filtered subcohort of 2,709 individuals with ASV-level taxa counts, BMF, sex, age, eGFR, and BMI data were selected. This filtering resulted in a total of 68 genera. For the metabolomics analysis, a cohort of 2,043 participants with BMF, sex, age, eGFR, BMI, and blood metabolomics data were selected. 973 metabolites were retained for downstream analyses. 274 blood proteins that met the prevalence filter in the cohort of 1,999 individuals were retained for downstream analyses. A similar prevalence filter was applied to 3,682 samples with blood plasma chemistries data, resulting in 68 features retained for downstream analyses. Similarly, for ordinal regression of the questionnaire data (e.g. diet, lifestyle, and stress/pain/health factors,) using the respective R package, polr [29], we collated all the responses and filtered out questions that contained more than 30% “NAs”. We also excluded binary responses, which are incompatible with ordinal regression using polr, which resulted in 277 variables across 2,291 participants, in addition to having paired data on age, sex, eGFR, BMI, and BMF. BMF data was captured from responses to a survey question on how many bowel movements an individual has per week, on average. The available responses to this question were: (1) Twice per week or less; (2) 3–6 times per week; (3) 1–3 times daily; or (4) 4 or more times daily. While the normal range of BMF encompasses both the second and third responses to this question (i.e., between three times a week and three times a day) [76], we chose to define 1–3 times per day (high-normal) as the reference group for the purposes of regression.

### Gut Microbiome Data

Fecal samples from Arivale participants were collected (described in Diener et al [12] and detailed here) from proprietary at-home kits developed by two microbiome vendors (DNA Genotek and Second Genome) that stabilize the DNA collected at ambient room temperature. Using the KingFisher Flex instrument, the MoBio PowerMag Soil DNA isolation kit (QIAGEN) enabled the isolation of stool DNA from 250 ml of homogenized human feces, after performing an additional glass bead-beating step. Qubit measurement and spectrophotometry were also performed using an A260/A280 absorbance ratio. Either 250-bp paired-end MiSeq profiling of the 16S V4 region (Second Genome, USA) or the 300-bp paired-end MiSeq profiling of the 16S V3-V4 region (DNA Genotek, USA) was used to obtain the raw amplicon sequencing data (ASVs).

16S amplicon sequencing was run on a MiSeq (Illumina, USA) with either paired-end 300-bp protocol (DNA Genotek) or paired-end 250-bp protocol (SecondGenome). The FASTQ files were provided by the Illumina Basespace platform after the phiX reads were removed with basecalling. Length cutoffs of 250-bp for the forward reads and 230-bp for the reverse reads as well as manual inspection of the error rate across sequencing cycles were determined from the respective profiles. Any greater than 2 expected errors under the Illumina error model resulted in eliminating that specific read from the data along with reads containing ambiguous (“N” nucleotides) base calls. Over 97% of the reads passed these filters, resulting in approximately 200,000 reads per sample.

Shotgun metagenomic sequencing libraries for Arivale samples were prepared by DNA Genotek using the NexteraXT kit, along with QC on a Bioanalyzer and quantification of DNA using qPCR for pooling. Sequencing was run on an Illumina NovaSeq6000 (300-multiplex on S2 flow cell), with a paired-end 150-bp protocol. The target sequencing depth was 3Gb, equivalent to about 20M total reads per sample.

Final truncated and filtered reads were then used to infer amplicon sequence variants (ASV) with DADA2. Each sequencing run separately resulted in its own error profiles. The final ASVs and counts were then joined, with chimeras being removed using DADA2’s “consensus” strategy. After this step, almost 16% of all reads were removed. Taxonomic assignment of ASVs was then achieved using the naive Bayes classifier in DADA2 with the SILVA database (version 128).

Wherever possible, the 16S gene in SILVA was used to perform by using an exact match of the inferred ASV to the gene. Nearly 90% of the reads were able to be classified down to the genus level, which was the taxonomic level chosen for this analysis. 3,694 samples across 609 taxa were available from these methods, which were then filtered down to 68 taxa after using a 70% prevalence filter (no more than 30% of data was permitted to be missing per filtered taxa). The diversity of the gut microbiomes of the cohort was characterized and rarefied to an even depth across ASVs where count parity is preserved across samples. Observed ASVs, a measure of species diversity, were used to obtain Shannon diversity and Pielou’s evenness. After BMI, sex, age, and eGFR data were merged to the taxa dataset, 2,709 samples remained across the 68 taxa.

The diversity of the gut microbiomes of the cohort was characterized and rarefied to an even depth (using the “rarefy_even_depth( )” function in the phyloseq R package [77]; rng seed = 111) with observed amplicon sequence variants (ASV), a measure of species diversity, to obtain Shannon diversity and Pielou’s evenness.

### Olink Proteomics

Blood plasma proteomic data were generated by Olink Biosciences using the ProSeek Cardiovascular II, Cardiovascular III, and Inflammation arrays. The proteins were filtered down to 274 proteins and 1,999 samples and included based on whether or not they had 30% or less missingness across samples as well as BMI, sex, age, and BMF data. NA data values were assumed to be below detection and imputed to be the median across samples for that particular protein. The values used for the proteomics analysis were from protein readings previously batch-corrected and normalized based on the overlapping reference samples within the batch plates. The corrected values were also scale-shifted to the reference sample and the original delivered data (using the seventh run as a baseline). The method is described further in the study by Zubair et al [78]. All data were merged with BMI, sex, age, and eGFR data for the cohort.

### Metabolon Metabolomics

Metabolon obtained metabolomics data on the previously mentioned plasma samples using preparation, quality control, and collection methods described in previous studies [50]. 2,043 total metabolites across 1,297 samples were filtered down using the same prevalence filter as for proteins. In this analysis, missing values were imputed to be the median of the non-missing samples for the metabolite, and final downstream metabolites were log-transformed and merged with available BMI, sex, age, and eGFR data.

### Blood Plasma Chemistries

LabCorp and Quest phlebotomists collected blood from Arivale participants within 21 days of their gut microbiome samples being taken, during the same blood draw as the metabolomics and using methods described previously by Wilmanski et al and others [12]. Individuals were asked to abstain from alcohol, vigorous exercise, monosodium glutamate and aspartame at least 24 hours prior to drawing of the blood, as well as fasting at least 12 hours beforehand. Blood samples were collected for blood plasma chemistries, metabolomics and proteomics at the same time, and within 21 days of stool sampling. BMI was calculated from weight and height using the following formula $BMI=\frac{weight\;(kg)}{{\left(height\;(m)\right)}^{2}}$. 4,881 samples and 127 laboratory values were filtered down using the same prevalence filtering as with metabolomics and proteomics. The final 68 features were log-transformed, with missing samples imputed to be the median value of the non-missing samples. These features were merged with other data and covariates. eGFR was calculated based on the CKD Epidemiology Collaboration (CKD-EPI) creatinine Equation (2021), as recommended by the current guidelines of the National Kidney Foundation [cite PMID: 34563581]: eGFR_cr_ = 142 × min(Scr/ , 1) × max(Scr/ , 1)^−1.200^ × 0.9938^Age^ × 1.012 [if female], where Scr = standardized serum creatinine in mg/dL, = 0.7 (female) or 0.9 (male), and = −0.241 (female) or −0.302 (male).

### Questionnaire Data

4,402 self-reported results to questionnaire data with 3,002 samples were retrieved from Arivale participants at the beginning of the study. After filtration, 283 downstream features remained, which were subsequently filtered down again to 182 question features by removing factored features with less than 10 responses per level and at least 2 nonmissing levels to the factor. Category responses were organized and numbered to be ordinally ascending in magnitude or intensity with relatively even-spaced differences in magnitude between categories wherever possible (i.e. for a factored feature with levels from 1,…,n, the level labeled “1” represented responses such as “Strongly Disagree”, “Never”, “None”, or the lowest frequency/intensity, and the level labeled “n” represented responses such as “Strongly Agree”, “Always”, or the greatest frequency/intensity). These features were merged with BMI, sex, age, and eGFR data available for this subcohort.

### Depression and Anxiety Health History Data

We used logistic regression to scrutinize associations between 11 (anxiety) and 10 (depression) independent binary (“true” or “false”) self-reported questions based on asking self-reported “self-current”, “self-past”, and “family” histories of depression or anxiety and BMF, with depression or anxiety encoded as a binary dependent variable, and BMF encoded as a categorical independent variable, and with the standard set of covariates (sex, age, BMI, and eGFR).

### Statistical Analyses

For the blood proteomics, plasma chemistries, and metabolite associations, generalized linear regression models were run using the LIMMA package in R [79]. BMF was encoded as a categorical variable (or in the case of analyzing alpha-diversity, it was also computed as an ordinal variable with a linear model coefficient) with categories: 1 = constipation (1–2 bowel movements per week), 2 = low-normal (3–6 bowel movements per week), 3 = high-normal (1–3 bowel movements per day), and 4 = diarrhea (4 or more bowel movements per day). In each regression covariates BMI, sex, age, and eGFR were included, in addition to BMF, to the response variable. The response variables were either: centered log ratio-transformed taxa data, log-transformed plasma metabolomics data, corrected plasma proteomics data, log-transformed plasma chemistries data, or ordinal response variables from questionnaire data, depending on the analysis. For gut microbiome data, genus-level counts were modeled with a beta-binomial distribution using the CORNCOB package in R [80]. Finally, for the questionnaire data (ordinal response categories across diet, exercise, stress, pain, and other lifestyle factors), the depression questions data, and the anxiety questions data, polr in R was used for the ordinal regression analysis.

### Community Replication Rate (PTR) of Gut Microbiome

FASTQ files from the metagenomic shotgun sequencing were first filtered and trimmed using FASTP. Here the first 5-bp of the 5’ end of the read were trimmed to remove partial adapter sequences and the 3’ end was trimmed using a sliding window that would trim the read as soon as the window average fell below a quality score of 20. Reads shorter than 50-bp after trimming or with more than 1 ambiguous base call were removed from further analysis. Filtered and trimmed reads were then passed to COPTR to estimate PTRs [81,82]. In brief, preprocessed reads were aligned to a database of 2,935 species representative genomes from the human gut contained in the IGG database version 1.0 using BOWTIE2. Coverage profiles were extracted from the generated alignments and log2-transformed PTRs were calculated by COPTR for each reference genome with at least 5,000 mapped reads. For each sample an overall measure of bacterial replication was estimated as the mean of all log2 PTR estimates in the sample. The mean log2 PTR was then used in a regression model as the dependent variable and regressed against BMF categories correcting for sex, age, and BMI. Significant associations with overall BMF were obtained from an F-test comparing the full model with a nested model containing only the confounding variables.

## Supplementary Material

Supplement 1

Supplement 2

## Data Availability

Code used to analyze 16S rRNA gene amplicon sequencing data can be found at https://github.com/gibbons-lab/mbtools while code used to run the statistical analysis described in this paper is available at https://github.com/jajohnso29/Generally-Healthy-Cohort-BMF . Pipelines for the processing of the metagenomic shotgun sequencing data and estimation of PTRs can be found at https://github.com/gibbons-lab/pipelines. Qualified researchers can access the full Arivale deidentified dataset, including all raw data, supporting the findings in this study for research purposes through signing a Data Use Agreement (DUA). Inquiries to access the data can be made at data-access@isbscience.org and will be responded to within 7 business days.

## What is already known about this topic:

Constipation and diarrhea are linked to several chronic diseases, like IBD, CKD, and neurodegenerative disorders. Chronic constipation, in particular, is associated with the increased production of microbially-derived uremic toxins in the gut due to an ecosystem-wide switch from fiber fermentation to protein fermentation. A build-up of these gut-derived toxins in blood, like p-cresol, has been associated with CKD disease progression and severity.

## What this study adds:

While prior work has demonstrated associations between microbially-derived uremic toxins, constipation, and CKD severity/progression, here we show similar signatures in a generally-healthy cohort. Overall, we map out the molecular phenotypic effects of aberrant BMFs across individuals without any apparent disease, and show how these effects precede, and may contribute to, the development of chronic disease. We find that certain lifestyle and dietary patterns, like higher levels of exercise, reduced anxiety levels, a more plant-based diet, and drinking more water, are associated with a more optimal BMF range.

## How this study might affect research, policy, or practice:

Overall, we suggest that even mild levels of chronic constipation may cause damage to organ systems over time and ultimately give rise to chronic diseases, like CKD or neurodegeneration. These findings pave the way for future research into early interventions for individuals at risk of developing chronic diseases related to BMF abnormalities. Managing BMF abnormalities prior to disease development may be an important disease prevention strategy, but this will require further evidence through longitudinal human intervention trials.
