# Supplementary material for: Generally-healthy individuals with aberrant bowel movement frequencies show enrichment for microbially-derived blood metabolites associated with reduced kidney function
Source: bioRxiv. 2024 Mar 9:2023.03.04.531100. Originally published 2023 Mar 6. Preprint. [Version 2] doi: 10.1101/2023.03.04.531100 (PMC10028848; doi:10.1101/2023.03.04.531100)
Supplement: 1 [file NIHPP2023.03.04.531100V2-supplement-1.pdf]

bioRxiv preprint doi: https://doi.org/10.1101/2023.03.04.531100; this version posted March 9, 2024. The copyright holder for this preprint (which was not certified by peer review) is the author/funder, who has granted bioRxiv a license to display the preprint in perpetuity. It is made available under aCC-BY 4.0 International license.

SUPPLEMENTAL FIGURES

**Covariates:**	**Mean ± standard deviation, or % across Arivale:**
Sex	65.1% Female
BMI	27.2 ± 5.89
Age	46.36 ± 12.96
eGFR	89.07 ± 20.20
CRP	2.40 ± 4.76
LDL	114.17 ± 33.77
A1C	5.49 ± 0.57
**Highlighted exclusionary criteria:**	
Percent with self-reported kidney disease	3.00% (119 out of 3,955 participants with BMF data available withheld from cohort)
Percent IBS or IBD	3.23% (128 out of 3,955 participants with BMF data available withheld from cohort)
**Exclusionary features (988 out of 3,955 participants with BMF data, or 25% of the initial BMF cohort, answered affirmatively to any of these and were excluded from the analyses. The final N of remaining participants after merging with covariates was N = 1,425 for the final baseline cohort):**	
Self - current history - bladder infection	
Self - current history - kidney disease	
Self - current history - kidney infection	
Self - current history - kidney stones	
Self - current history - bladder/kidney - other	
Self - current history - polycystic kidney disease (PKD)	
Self - current history - urinary incontinence	
Self - current history - kidney cancer	
Self - current history - celiac disease	
Self - current history - colonic Crohn's disease	
Self - current history - diverticulosis	
Self - current history - gastroesophageal reflux disease (GERD)	
Self - current history - ileal Crohn's disease	
Self - current history - irritable bowel syndrome (IBS)	
Self - current history - inflammatory bowel disease (IBD)	
Self - current history - ulcerative colitis	
Self - current history - peptic ulcer	
Self - laxatives usage	
Self - anticoagulation or cholesterol drugs usage	
Self - blood pressure drugs usage	

Figure S1. The modeling covariates and exclusionary criteria. Out of the 3,955 total Arivale participants that had BMF data, 3.00% self-reported kidney disease (the kidney-related questions in the exclusionary features) and 3.23% self-reported IBS or IBD. An initial baseline cohort of 3,132 participants that had health history survey questionnaire data was available. The participants that answered affirmatively to the exclusionary features were removed from the analysis, resulting in 25% of the initial cohort with BMF data being filtered down to N = 1,561, and subsequently, a final baseline cohort of 1,425 individuals after merging for covariates.

bioRxiv preprint doi: https://doi.org/10.1101/2023.03.04.531100; this version posted March 9, 2024. The copyright holder for this preprint (which was not certified by peer review) is the author/funder, who has granted bioRxiv a license to display the preprint in perpetuity. It is made available under aCC-BY 4.0 International license.

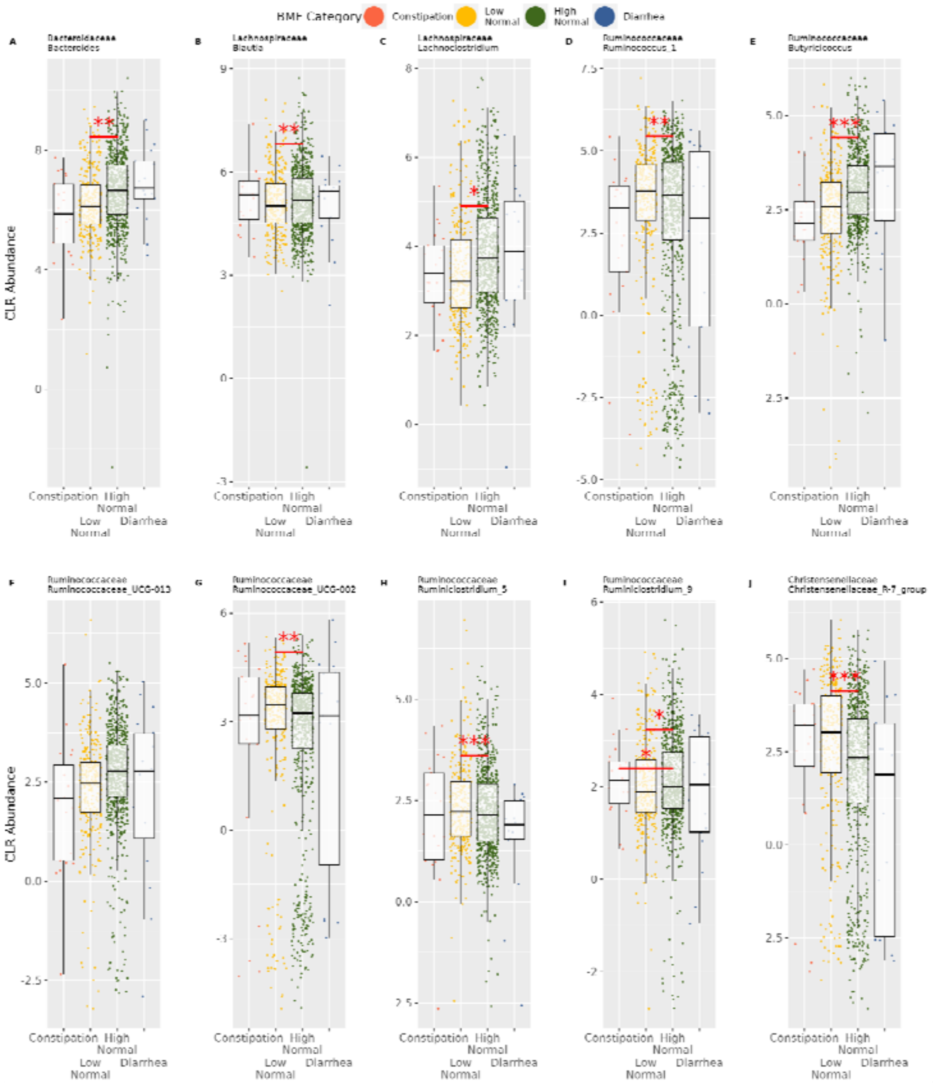

Low BMF ↔ High BMF

bioRxiv preprint doi: https://doi.org/10.1101/2023.03.04.531100; this version posted March 9, 2024. The copyright holder for this preprint (which was not certified by peer review) is the author/funder, who has granted bioRxiv a license to display the preprint in perpetuity. It is made available under aCC-BY 4.0 International license.

Figure S2. The top 10 most abundant genera significantly associated with BMF (A-J). Significant genera from the CORNCOB analysis in order of decreasing CLR-transformed abundance. The line in each plot denotes significant differences from the reference category ("High Normal" BMF), and asterisks denote FDR-corrected significance threshold. (***): $p < 0.0001$, (**): $0.0001 < p < 0.01$, (*): $0.01 < p < 0.05$. The horizontal axes are annotated as four BMF categories: "Constipation" (BMF = 1-2× per week), "Low Normal" (BMF = 3-6× per week), "High Normal" (BMF = 1-3× per day) which is the reference category in regression, and "Diarrhea" (BMF = 4× or more per day).

bioRxiv preprint doi: https://doi.org/10.1101/2023.03.04.531100; this version posted March 9, 2024. The copyright holder for this preprint (which was not certified by peer review) is the author/funder, who has granted bioRxiv a license to display the preprint in perpetuity. It is made available under aCC-BY 4.0 International license.

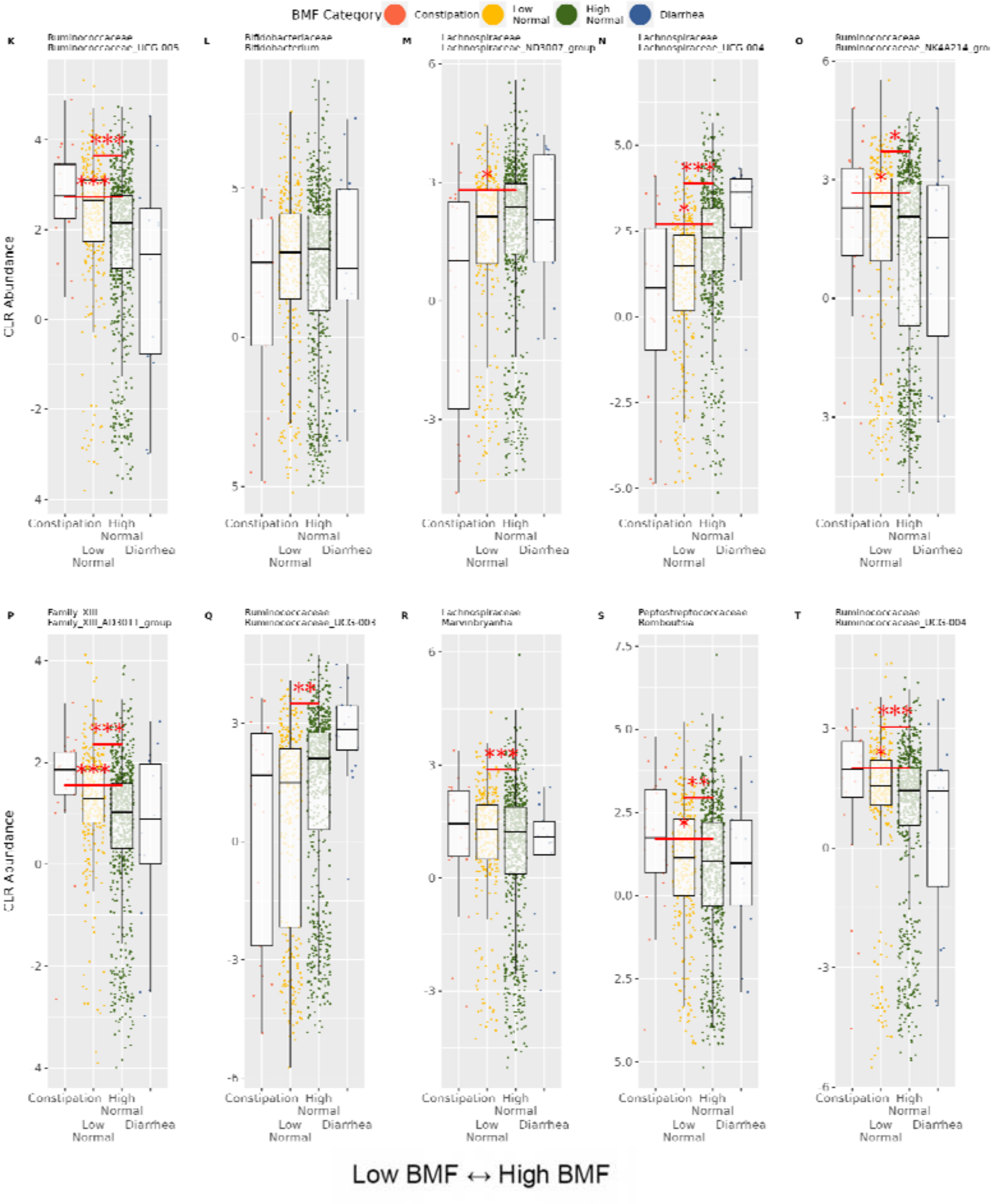

bioRxiv preprint doi: https://doi.org/10.1101/2023.03.04.531100; this version posted March 9, 2024. The copyright holder for this preprint (which was not certified by peer review) is the author/funder, who has granted bioRxiv a license to display the preprint in perpetuity. It is made available under aCC-BY 4.0 International license.

Figure S3. The top 11-20 most abundant genera associated with BMF (K-T). Significant genera from the CORNCOB analysis in order of decreasing CLR-transformed abundance. The line in each plot denotes significant differences from the reference category ("High Normal" BMF), and asterisks denote FDR-corrected significance threshold. (***): $p < 0.0001$, (**): $0.0001 < p < 0.01$, (*): $0.01 < p < 0.05$. The horizontal axes are annotated as four BMF categories: "Constipation" (BMF = 1-2× per week), "Low Normal" (BMF = 3-6× per week), "High Normal" (BMF = 1-3× per day) which is the reference category in regression, and "Diarrhea" (BMF = 4× or more per day).

bioRxiv preprint doi: https://doi.org/10.1101/2023.03.04.531100; this version posted March 9, 2024. The copyright holder for this preprint (which was not certified by peer review) is the author/funder, who has granted bioRxiv a license to display the preprint in perpetuity. It is made available under aCC-BY 4.0 International license.

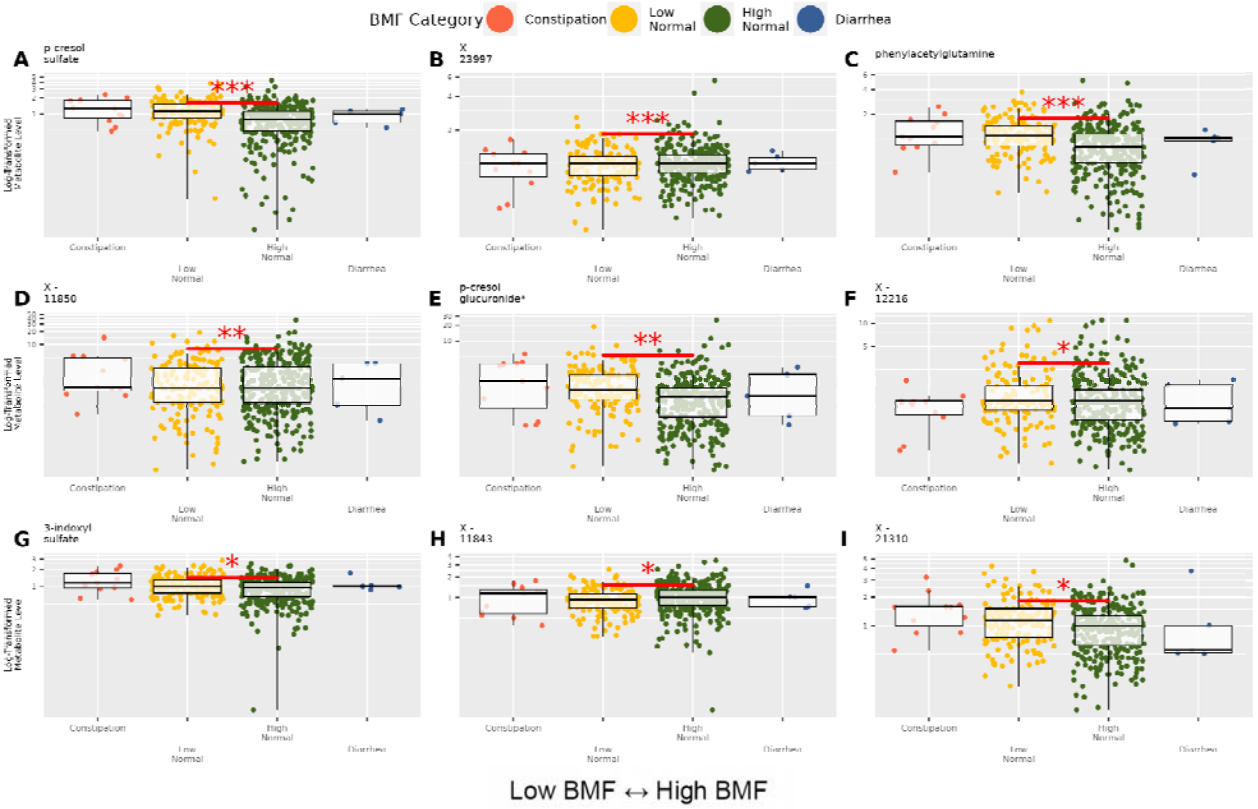

Figure S4. Significant BMF-associated plasma metabolites boxplots (A-I). Significant plasma metabolites from the LIMMA analysis. The horizontal axes are annotated as four BMF categories: “Constipation” (BMF = 1-2× per week), “Low Normal” (BMF = 3-6× per week), “High Normal” (BMF = 1-3× per day) which is the reference category in regression, and “Diarrhea” (BMF = 4× or more per day). Red significant comparison lines across each plot denote significant differences from the reference category (“High Normal” BMF), and asterisks denote FDR-corrected significance threshold. (***): $p < 0.0001$, (**): $0.0001 < p < 0.01$, (*): $0.01 < p < 0.05$.

bioRxiv preprint doi: https://doi.org/10.1101/2023.03.04.531100; this version posted March 9, 2024. The copyright holder for this preprint (which was not certified by peer review) is the author/funder, who has granted bioRxiv a license to display the preprint in perpetuity. It is made available under aCC-BY 4.0 International license.

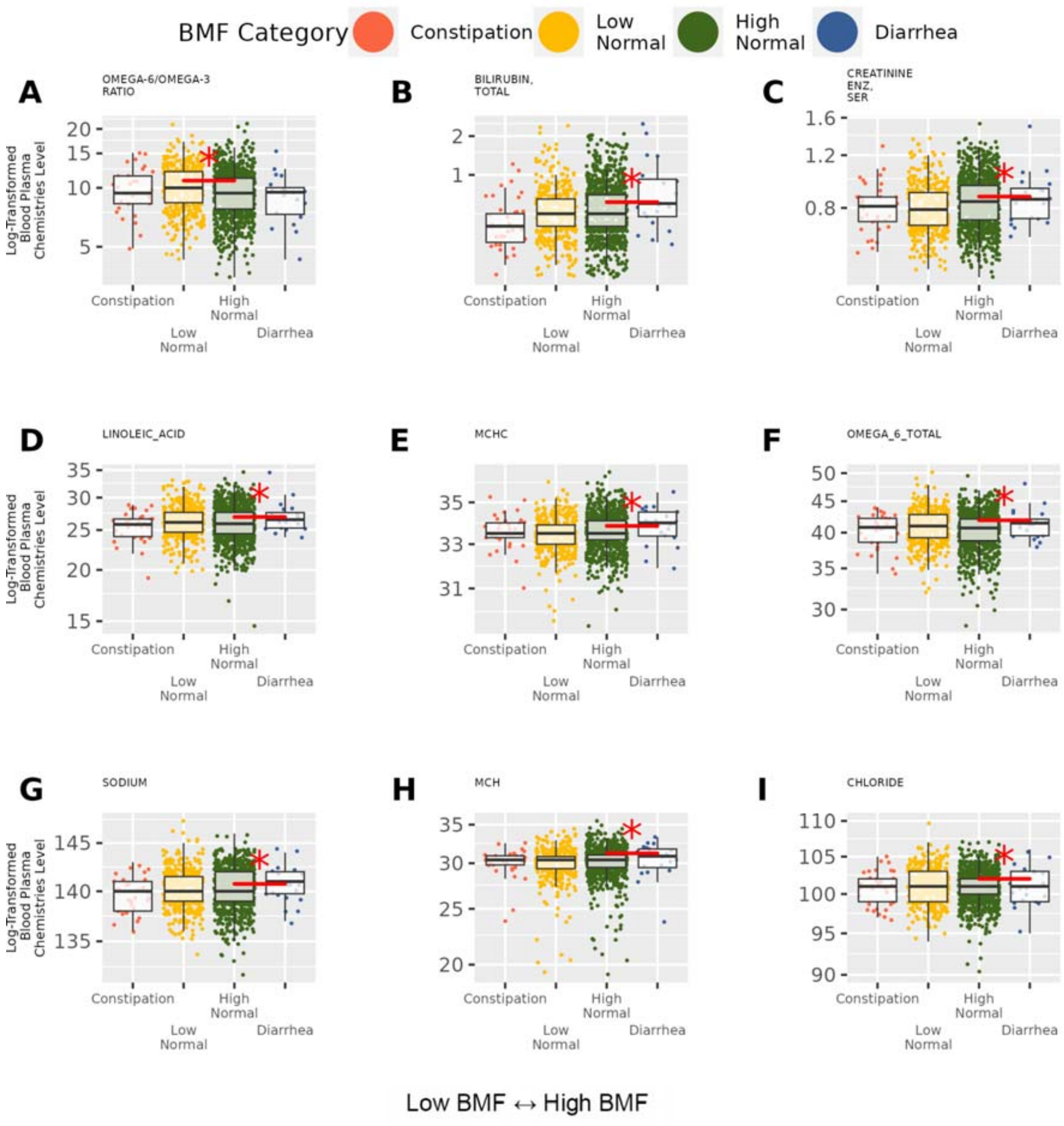

bioRxiv preprint doi: https://doi.org/10.1101/2023.03.04.531100; this version posted March 9, 2024. The copyright holder for this preprint (which was not certified by peer review) is the author/funder, who has granted bioRxiv a license to display the preprint in perpetuity. It is made available under aCC-BY 4.0 International license.

Figure S5. Significant BMF-associated clinical chemistries boxplots (A-I). Significant clinical chemistries from the LIMMA analysis. The horizontal axes are annotated as four BMF categories: “Constipation” (BMF = 1-2× per week), “Low Normal” (BMF = 3-6× per week), “High Normal” (BMF = 1-3× per day) which is the reference category in regression, and “Diarrhea” (BMF = 4× or more per day). Red significant comparison lines across each plot denote significant differences from the reference category (“High Normal” BMF), and asterisks denote FDR-corrected significance threshold. (***): $p < 0.0001$, (**): $0.0001 < p < 0.01$, (*): $0.01 < p < 0.05$.

bioRxiv preprint doi: https://doi.org/10.1101/2023.03.04.531100; this version posted March 9, 2024. The copyright holder for this preprint (which was not certified by peer review) is the author/funder, who has granted bioRxiv a license to display the preprint in perpetuity. It is made available under aCC-BY 4.0 International license.

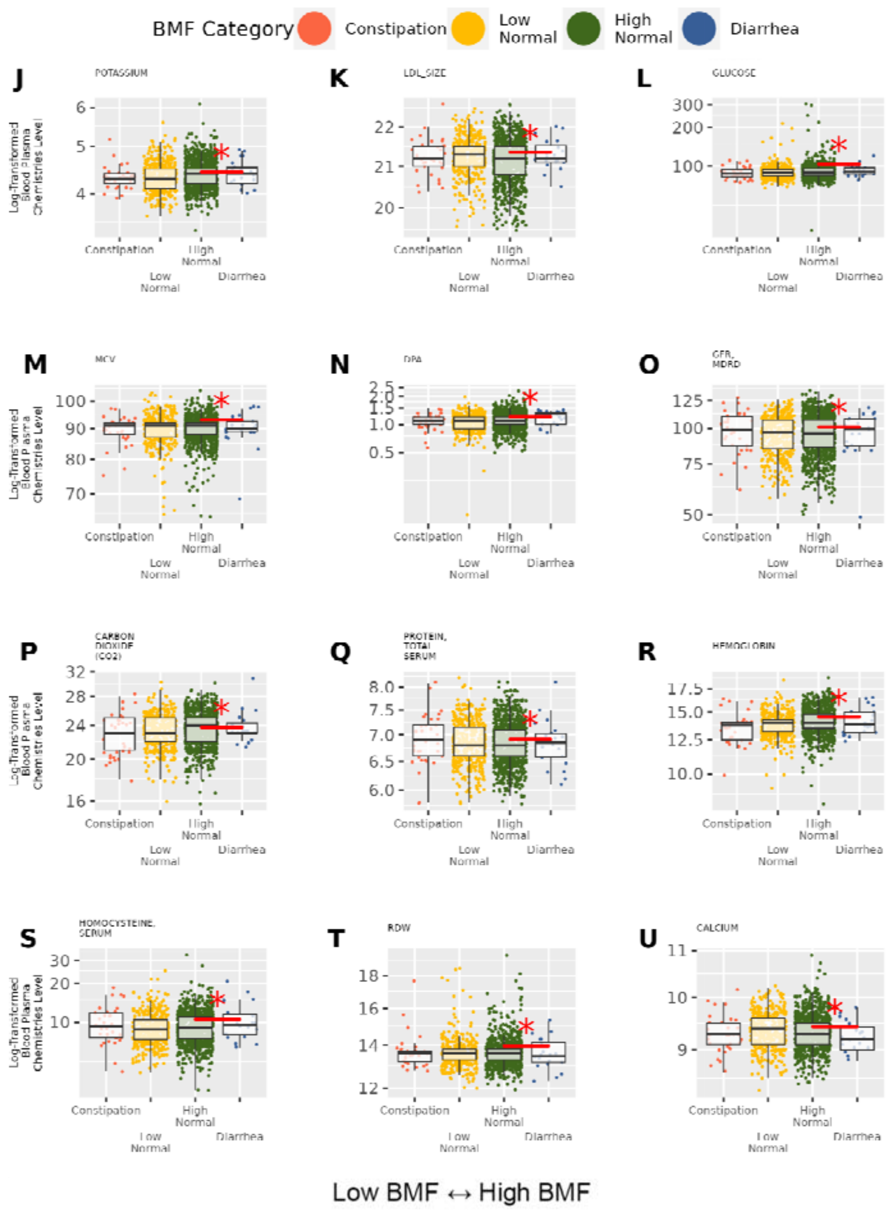

bioRxiv preprint doi: https://doi.org/10.1101/2023.03.04.531100; this version posted March 9, 2024. The copyright holder for this preprint (which was not certified by peer review) is the author/funder, who has granted bioRxiv a license to display the preprint in perpetuity. It is made available under aCC-BY 4.0 International license.

Figure S6. The remaining significant BMF-associated clinical chemistries boxplots (J-U).

The remaining significant clinical chemistries from the LIMMA analysis. The horizontal axes are annotated as four BMF categories: “Constipation” (BMF = 1-2× per week), “Low Normal” (BMF = 3-6× per week), “High Normal” (BMF = 1-3× per day) which is the reference category in regression, and “Diarrhea” (BMF = 4× or more per day). Red significant comparison lines across each plot denote significant differences from the reference category (“High Normal” BMF), and asterisks denote FDR-corrected significance threshold. (***): $p < 0.0001$, (**): $0.0001 < p < 0.01$, (*): $0.01 < p < 0.05$.

bioRxiv preprint doi: https://doi.org/10.1101/2023.03.04.531100; this version posted March 9, 2024. The copyright holder for this preprint (which was not certified by peer review) is the author/funder, who has granted bioRxiv a license to display the preprint in perpetuity. It is made available under aCC-BY 4.0 International license.

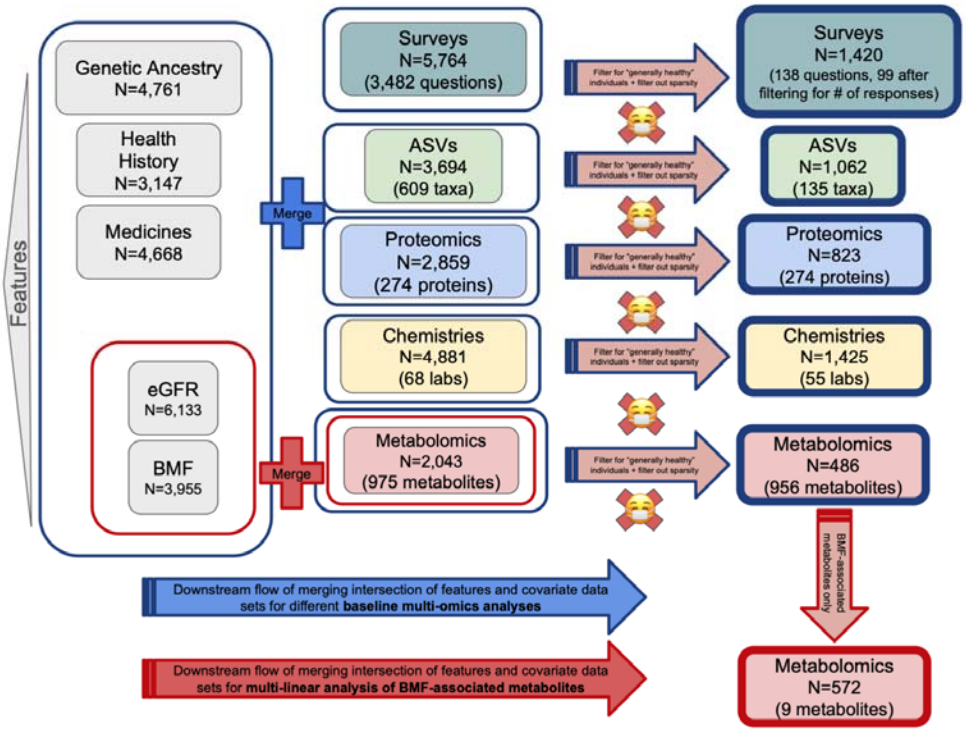

bioRxiv preprint doi: https://doi.org/10.1101/2023.03.04.531100; this version posted March 9, 2024. The copyright holder for this preprint (which was not certified by peer review) is the author/funder, who has granted bioRxiv a license to display the preprint in perpetuity. It is made available under aCC-BY 4.0 International license.

Figure S7. Flow Chart for Cohort Selection of Baseline Population. Individuals with the full complement of covariate data (sex, age, BMI, and CRP, LDL, A1C, and PCs1-3) were further filtered for having available baseline data for each of the following: surveys, microbiome profiles, proteomics, clinical chemistries (e.g. complete blood count, or CBC; and comprehensive metabolic panel, or CMP) and metabolomics. The "generally-healthy" exclusion criteria were then imposed (38.5% excluded; see Method Details), along with sparsity or non-missingness minimums for the features in the 'omics data (≥ 30% prevalence for gut microbiome data, metabolomics and clinical chemistries; ≥ 50% prevalence for proteomics; and ≥ 90% prevalence and ≥ 10% affirmative for binary responses in the survey questions). These filters resulted in the final sub-cohort numbers shown on the right side of the figure in blue outlines. Additionally, the eGFR and BMF data frames were merged with the metabolomics data frame and filtered by the "generally-healthy" exclusionary criteria to achieve 572 participants with the data for the 9 BMF-associated metabolites eGFR regression and mediation analysis.

bioRxiv preprint doi: https://doi.org/10.1101/2023.03.04.531100; this version posted March 9, 2024. The copyright holder for this preprint (which was not certified by peer review) is the author/funder, who has granted bioRxiv a license to display the preprint in perpetuity. It is made available under aCC-BY 4.0 International license.

```
                            OLS Regression Results
==============================================================================
Dep. Variable:                   eGFR   R-squared:                       0.082
Model:                            OLS   Adj. R-squared:                  0.067
Method:                 Least Squares   F-statistic:                     5.547
Date:                Sun, 18 Feb 2024   Prob (F-statistic):           2.42e-07
Time:                        07:29:22   Log-Likelihood:                -2465.4
No. Observations:                 572   AIC:                             4951.
Df Residuals:                     562   BIC:                             4994.
Df Model:                           9
Covariance Type:            nonrobust
=========================================================================================
                            coef    std err          t      P>|t|      [0.025      0.975]
-----------------------------------------------------------------------------------------
const                   115.0755      4.841     23.770      0.000     105.566     124.585
bowel                    -3.9902      1.496     -2.667      0.008      -6.929      -1.051
p-cresol sulfate         -2.6898      2.473     -1.088      0.277      -7.548       2.168
X - 23997                 1.7076      1.423      1.200      0.231      -1.087       4.502
phenylacetylglutamine     2.2073      2.247      0.982      0.326      -2.207       6.622
X - 11850                -0.4421      0.359     -1.232      0.218      -1.147       0.263
p-cresol glucuronide      0.3677      0.490      0.750      0.454      -0.595       1.330
X - 12216                -1.9827      0.787     -2.520      0.012      -3.528      -0.437
3-indoxyl sulfate        -9.6859      2.249     -4.307      0.000     -14.104      -5.268
X - 11843                 0.0527      0.554      0.095      0.924      -1.036       1.141
==============================================================================
Omnibus:                       39.704   Durbin-Watson:                   1.841
Prob(Omnibus):                  0.000   Jarque-Bera (JB):               22.585
Skew:                          -0.333   Prob(JB):                     1.25e-05
Kurtosis:                       2.290   Cond. No.                         45.4
==============================================================================
```

Figure S8. OLS regression resulting from eGFR ~ BMF-associated metabolites + BMF. The p-value for the overall generalized-linear model (eGFR ~ BMF-related metabolites) was significant (N = 572, p = 2.42E-7, R^2 = 0.082) and so were the p-values of the individual β-coefficients for 3-IS ($\beta_{3\text{-IS}}$ = -9.69, p = 1.96E-5), BMF (denoted “bowel”; β_{BMF} = -3.99, p = 7.88E-3), and X - 12216 ($\beta_{X\text{ - }12216}$ = -1.98, p = 1.20E-2).
