# Supplementary material for: Generally-healthy individuals with aberrant bowel movement frequencies show enrichment for microbially-derived blood metabolites associated with impaired kidney function
Source: bioRxiv. 2023 Mar 6:2023.03.04.531100. Preprint. [Version 1] doi: 10.1101/2023.03.04.531100 (PMC10028848; doi:10.1101/2023.03.04.531100)
Supplement: Supplementary file 1 [file media-1.pdf]

Appendix D: Current Assessment questions

Assessment	Question Text	Options
Diet	Do you follow a specific dietary plan?	['No special diet', 'Vegetarian', 'Vegan', 'Gluten-free', 'Paleo', 'Weight loss (e.g. Weight Watchers, Jenny Craig)', 'Low carbohydrate (Atkins, South Beach, Zone, etc.)', 'Diabetic', 'Other']
Diet	What kind of bread do you usually eat?	['White', 'Wheat (not whole wheat)', 'Whole grain', 'Other kind', 'No usual type', 'Do not know', 'Do not eat bread']
Diet	What do you usually spread on bread?	['Butter', 'Margarine', 'Low fat spread', 'Other', 'No usual

©2017-2018 Arivale Inc. All Rights Reserved. Confidential and Proprietary.

		type', 'Do not know', 'Do not spread fat on bread']
Diet	How many slices of bread/rolls do you usually eat in a day?	['Less than 1', '1', '2-3', '4-5', '6 or more']
Diet	How many cookies, cakes, scones, sweet pies, and pastries do you usually eat in a day?	['Less than 1', '1', '2-3', '4-5', '6 or more']
Diet	What kind of milk do you usually use for drinks in tea or coffee and on cereals etc?	['Whole milk', '1-2%', 'Skimmed', 'Other kind', 'No usual type', 'Do not know', 'Do not drink milk']
Diet	Do you usually take sugar in tea?	["I don't drink tea", 'No', 'Yes']
Diet	Do you usually take sugar in coffee?	["I don't drink coffee", 'No', 'Yes']
Diet	Do you:	['Generally add salt to food without tasting first', 'Taste food and then generally add salt', 'Taste food but only occasionally add salt', 'Rarely or never add salt to food']
Diet	Which type of breakfast cereal do you normally eat?	['High fiber (e.g. All Bran, Bran flakes, Shredded wheat, Muesli, Oatmeal, Weetabix)', 'Other (e.g. Corn flakes, Rice Krispies, Special K, Sugar Puffs)', 'No usual type', 'Do not eat breakfast cereal']
Diet	Breakfast cereal	['Less than once per month', '1-3 times per month', 'Once per week', '2-4 times per week', '5-6 times per week', 'Once per day', '2-3 times per day', '4-5 times per day', '6+ times per day']
Diet	Fresh fruit	['Less than once per month', '1-3 times per month', 'Once per week', '2-4 times per week', '5-6 times per week', 'Once per day', '2-3 times per day', '4-5 times per day', '6+ times per day']

©2017-2018 Arivale Inc. All Rights Reserved. Confidential and Proprietary.

Diet	Cooked green vegetables (fresh or frozen)	['Less than once per month', '1-3 times per month', 'Once per week', '2-4 times per week', '5-6 times per week', 'Once per day', '2-3 times per day', '4-5 times per day', '6+ times per day']
Diet	Cooked root vegetables (fresh or frozen)	['Less than once per month', '1-3 times per month', 'Once per week', '2-4 times per week', '5-6 times per week', 'Once per day', '2-3 times per day', '4-5 times per day', '6+ times per day']
Diet	Raw vegetables or salad (including tomatoes)	['Less than once per month', '1-3 times per month', 'Once per week', '2-4 times per week', '5-6 times per week', 'Once per day', '2-3 times per day', '4-5 times per day', '6+ times per day']
Diet	French fries	['Less than once per month', '1-3 times per month', 'Once per week', '2-4 times per week', '5-6 times per week', 'Once per day', '2-3 times per day', '4-5 times per day', '6+ times per day']
Diet	Potatoes, pasta, or rice	['Less than once per month', '1-3 times per month', 'Once per week', '2-4 times per week', '5-6 times per week', 'Once per day', '2-3 times per day', '4-5 times per day', '6+ times per day']
Diet	Meat (beef, lamb, or pork)	['Less than once per month', '1-3 times per month', 'Once per week', '2-4 times per week', '5-6 times per week', 'Once per day', '2-3 times per day', '4-5 times per day', '6+ times per day']
Diet	Processed meat (e.g. bacon, sausage, hot dogs, etc.)	['Less than once per month', '1-3 times per month', 'Once per week', '2-4 times per week', '5-6 times per week',

©2017-2018 Arivale Inc. All Rights Reserved. Confidential and Proprietary.

		'Once per day', '2-3 times per day', '4-5 times per day', '6+ times per day']
Diet	Poultry (e.g. chicken, turkey, duck, etc.)	['Less than once per month', '1-3 times per month', 'Once per week', '2-4 times per week', '5-6 times per week', 'Once per day', '2-3 times per day', '4-5 times per day', '6+ times per day']
Diet	White fish (e.g. cod, halibut, flounder, etc.)	['Less than once per month', '1-3 times per month', 'Once per week', '2-4 times per week', '5-6 times per week', 'Once per day', '2-3 times per day', '4-5 times per day', '6+ times per day']
Diet	Oil rich fish (e.g. salmon, tuna, anchovies, etc.)	['Less than once per month', '1-3 times per month', 'Once per week', '2-4 times per week', '5-6 times per week', 'Once per day', '2-3 times per day', '4-5 times per day', '6+ times per day']
Diet	Cheese	['Less than once per month', '1-3 times per month', 'Once per week', '2-4 times per week', '5-6 times per week', 'Once per day', '2-3 times per day', '4-5 times per day', '6+ times per day']
Diet	Beans or legumes (e.g. lentils, peas, pinto beans, etc.)	['Less than once per month', '1-3 times per month', 'Once per week', '2-4 times per week', '5-6 times per week', 'Once per day', '2-3 times per day', '4-5 times per day', '6+ times per day']
Diet	Sweets or chocolates	['Less than once per month', '1-3 times per month', 'Once per week', '2-4 times per week', '5-6 times per week', 'Once per day', '2-3 times per day', '4-5 times per day', '6+ times per day']

©2017-2018 Arivale Inc. All Rights Reserved. Confidential and Proprietary.

Diet	Ice cream	['Less than once per month', '1-3 times per month', 'Once per week', '2-4 times per week', '5-6 times per week', 'Once per day', '2-3 times per day', '4-5 times per day', '6+ times per day']
Diet	Crackers, pretzels, chips, or savory snacks	['Less than once per month', '1-3 times per month', 'Once per week', '2-4 times per week', '5-6 times per week', 'Once per day', '2-3 times per day', '4-5 times per day', '6+ times per day']
Diet	100% fruit juice	['Less than once per month', '1-3 times per month', 'Once per week', '2-4 times per week', '5-6 times per week', 'Once per day', '2-3 times per day', '4-5 times per day', '6+ times per day']
Diet	Soft drinks/soda	['Less than once per month', '1-3 times per month', 'Once per week', '2-4 times per week', '5-6 times per week', 'Once per day', '2-3 times per day', '4-5 times per day', '6+ times per day']
Diet	Cakes, scones, sweet pies, or pastries	['Less than once per month', '1-3 times per month', 'Once per week', '2-4 times per week', '5-6 times per week', 'Once per day', '2-3 times per day', '4-5 times per day', '6+ times per day']
Diet	Water (e.g. 8 oz. cups)	['Less than once per month', '1-3 times per month', 'Once per week', '2-4 times per week', '5-6 times per week', 'Once per day', '2-3 times per day', '4-5 times per day', '6+ times per day']

©2017-2018 Arivale Inc. All Rights Reserved. Confidential and Proprietary.

Diet	Alcoholic beverages (e.g. 12 oz. of beer, 5 oz. of wine, 1.5 oz. shot of liquor)	['Less than once per month', '1-3 times per month', 'Once per week', '2-4 times per week', '5-6 times per week', 'Once per day', '2-3 times per day', '4-5 times per day', '6+ times per day']
Diet	What type of alcohol do you most often drink?	['Beer', 'Wine', 'Liquor', 'Other kind', 'No usual type', 'I do not know', 'I do not drink alcohol']
Diet	How many drinks do you have on a drinking day?	[‘I do no drink’, ‘1-2 drinks’, ‘3-4 drinks’, ‘5-6 drinks’, ‘More than 6 drinks’]
Diet	How many servings of sugary drinks (including soda, energy drinks, sweetened tea or coffee, etc) do you have each day?	[‘Zero/less than 1 per day’, ‘1’, ‘2-3, ‘4-5’, ‘6 or more’]
Diet	Yogurt with live cultures	['Less than once per month', '1-3 times per month', 'Once per week', '2-4 times per week', '5-6 times per week', 'Once per day', '2-3 times per day', '4-5 times per day', '6+ times per day']
Diet	Eggs	['Less than once per month', '1-3 times per month', 'Once per week', '2-4 times per week', '5-6 times per week', 'Once per day', '2-3 times per day', '4-5 times per day', '6+ times per day']
Diet	Eat food prepared by someone outside of your home (e.g. dining out at a restaurant or take-out)	['Less than once per month', '1-3 times per month', 'Once per week', '2-4 times per week', '5-6 times per week', 'Once per day', '2-3 times per day', '4-5 times per day', '6+ times per day']
Diet	Cook meals at home	['Less than once per month', '1-3 times per month', 'Once per week', '2-4 times per week', '5-6 times per week', 'Once per day', '2-3 times per day', '4-5 times per day', '6+ times per day']

©2017-2018 Arivale Inc. All Rights Reserved. Confidential and Proprietary.

Arivale
Your scientific path to wellness

Diet	Eat breakfast	['Less than once per month', '1-3 times per month', 'Once per week', '2-4 times per week', '5-6 times per week', 'Once per day', '2-3 times per day', '4-5 times per day', '6+ times per day']
Diet	Snack between meals	['Less than once per month', '1-3 times per month', 'Once per week', '2-4 times per week', '5-6 times per week', 'Once per day', '2-3 times per day', '4-5 times per day', '6+ times per day']
Diet	Plant based protein (e.g. tofu, beans, lentils, tempeh)	['Less than once per month', '1-3 times per month', 'Once per week', '2-4 times per week', '5-6 times per week', 'Once per day', '2-3 times per day', '4-5 times per day', '6+ times per day']
Diet	Nuts/seeds (e.g. almonds, walnuts, flax seeds, etc.)	['Less than once per month', '1-3 times per month', 'Once per week', '2-4 times per week', '5-6 times per week', 'Once per day', '2-3 times per day', '4-5 times per day', '6+ times per day']
Diet	Cruciferous vegetables (e.g. broccoli, cabbage, cauliflower, etc.)	['Less than once per month', '1-3 times per month', 'Once per week', '2-4 times per week', '5-6 times per week', 'Once per day', '2-3 times per day', '4-5 times per day', '6+ times per day']
Diet	How many servings of vegetables (including juice) do you have each day? (1 serving = 1 cup raw/leafy vegetables, ½ cup cooked vegetables, or ½ cup vegetable juice)	['Zero/less than 1 per day', '1', '2-3, '4-5', '6 or more']
Diet	How many servings of fruit (including juice) do you have each day? (1 serving = 1 medium piece of whole fruit,	['Zero/less than 1 per day', '1', '2-3, '4-5', '6 or more']

©2017-2018 Arivale Inc. All Rights Reserved. Confidential and Proprietary.

	½ cup sliced fruit/berries or ½ cup juice)	
Diet	How many servings of whole grains do you have each day (1 serving = 1 slice of whole grain bread, ½ cup cooked oats, brown rice, whole wheat pasta, etc.)?	['Zero/less than 1 per day', '1-2', '3-4, '5-6', '7 or more']
Diet	Caffeinated beverages (e.g. 8 oz. cup of coffee or black tea, one shot of espresso, one energy drink)	['Less than once per month', '1-3 times per month', 'Once per week', '2-4 times per week', '5-6 times per week', 'Once per day', '2-3 times per day', '4-5 times per day', '6+ times per day']
Diet	Fermented foods (e.g. sauerkraut, kombucha, kimchi, etc.)	['Less than once per month', '1-3 times per month', 'Once per week', '2-4 times per week', '5-6 times per week', 'Once per day', '2-3 times per day', '4-5 times per day', '6+ times per day']
Diet	If you specified other dietary plan, please describe.	[None]
Diet	Do you avoid dairy products?	['No', 'Yes']
Digestion	I experience nausea.	['Always', 'Often (3 times or more per week)', 'Seldom (1-2 times per week)', 'Never']
Digestion	I have acid reflux/heartburn.	['Daily', 'More than 1 time per week', 'Less than weekly', 'Not at all']
Digestion	I have bloating.	['Daily', 'Several times per week', 'Once per week or less', 'Not at all']
Digestion	I have loose stools or diarrhea.	['Daily', 'Several times per week', 'Once per week or less', 'Not at all']
Digestion	Do you have a high appetite and/or overeat?	['Regularly (daily or several times per week)', 'Occasionally (once a week or less)', 'No']
Digestion	I experience vomiting	['Regularly (daily or several times per week)', 'Occasionally (once a week or less)', 'Infrequently or not at all (less than monthly)']

©2017-2018 Arivale Inc. All Rights Reserved. Confidential and Proprietary.

Digestion	I have abdominal/digestive pain	['Daily', 'More than 1 time per week', 'Less than weekly', 'Not at all']
Digestion	I experience gas.	['Daily', 'Several times per week', 'Once per week or less', 'Not at all']
Digestion	I have bowel movements	['2 or fewer times per week', '3-6 times per week', '1-3 times daily', '4+ times daily']
Digestion	My bowel movements are	['Often difficult', 'Sometimes difficult', 'Easy to pass']
Digestion	My bowel movements feel complete	['Infrequently', 'Sometimes', 'Most of the time']
Digestion	I take probiotic supplements/eat fermented foods	['Daily', 'Several times per week', 'Once per week or less', 'Not at all']
Digestion	I take laxatives	['Daily', 'Several times per week', 'Once per week or less', 'Not at all']
Digestion	I take medications (over the counter or prescription) for heartburn or acid reflux	['Daily', 'Several times per week', 'Once per week or less', 'Not at all']
Digestion	I eat under stress	['Daily', 'Several times per week', 'Once per week or less', 'Not at all']
Digestion	Do you have a poor appetite and/or skip meals?	['Regularly (daily or several times per week)', 'Occasionally (once a week or less)', 'No']
Happiness	I don't feel particularly pleased with the way I am.	['Strongly Agree', 'Moderately Agree', 'Slightly Agree', 'Slightly Disagree', 'Moderately Disagree', 'Strongly Disagree']
Happiness	I am intensely interested in other people.	['Strongly Disagree', 'Moderately Disagree', 'Slightly Disagree', 'Slightly Agree', 'Moderately Agree', 'Strongly Agree']
Happiness	I feel that life is very rewarding.	['Strongly Disagree', 'Moderately Disagree', 'Slightly Disagree', 'Slightly Agree', 'Moderately Agree', 'Strongly Agree']
Happiness	I have very warm feelings towards almost everyone.	['Strongly Disagree', 'Moderately Disagree',

©2017-2018 Arivale Inc. All Rights Reserved. Confidential and Proprietary.

		'Slightly Disagree', 'Slightly Agree', 'Moderately Agree', 'Strongly Agree']
Happiness	I rarely wake up feeling rested.	['Strongly Agree', 'Moderately Agree', 'Slightly Agree', 'Slightly Disagree', 'Moderately Disagree', 'Strongly Disagree']
Happiness	I am not particularly optimistic about the future.	['Strongly Agree', 'Moderately Agree', 'Slightly Agree', 'Slightly Disagree', 'Moderately Disagree', 'Strongly Disagree']
Happiness	I find most things amusing.	['Strongly Disagree', 'Moderately Disagree', 'Slightly Disagree', 'Slightly Agree', 'Moderately Agree', 'Strongly Agree']
Happiness	I am always committed and involved.	['Strongly Disagree', 'Moderately Disagree', 'Slightly Disagree', 'Slightly Agree', 'Moderately Agree', 'Strongly Agree']
Happiness	Life is good.	['Strongly Disagree', 'Moderately Disagree', 'Slightly Disagree', 'Slightly Agree', 'Moderately Agree', 'Strongly Agree']
Happiness	I do not think that the world is a good place.	['Strongly Agree', 'Moderately Agree', 'Slightly Agree', 'Slightly Disagree', 'Moderately Disagree', 'Strongly Disagree']
Happiness	I laugh a lot.	['Strongly Disagree', 'Moderately Disagree', 'Slightly Disagree', 'Slightly Agree', 'Moderately Agree', 'Strongly Agree']
Happiness	I am well satisfied about everything in my life.	['Strongly Disagree', 'Moderately Disagree', 'Slightly Disagree', 'Slightly Agree', 'Moderately Agree', 'Strongly Agree']
Happiness	I don't think I look attractive.	['Strongly Agree', 'Moderately Agree', 'Slightly Agree',

©2017-2018 Arivale Inc. All Rights Reserved. Confidential and Proprietary.

		'Slightly Disagree', 'Moderately Disagree', 'Strongly Disagree']
Happiness	There is a gap between what I would like to do and what I have done.	['Strongly Agree', 'Moderately Agree', 'Slightly Agree', 'Slightly Disagree', 'Moderately Disagree', 'Strongly Disagree']
Happiness	I am very happy.	['Strongly Disagree', 'Moderately Disagree', 'Slightly Disagree', 'Slightly Agree', 'Moderately Agree', 'Strongly Agree']
Happiness	I find beauty in some things.	['Strongly Disagree', 'Moderately Disagree', 'Slightly Disagree', 'Slightly Agree', 'Moderately Agree', 'Strongly Agree']
Happiness	I always have a cheerful effect on others.	['Strongly Disagree', 'Moderately Disagree', 'Slightly Disagree', 'Slightly Agree', 'Moderately Agree', 'Strongly Agree']
Happiness	I can fit in (find time for) everything I want to.	['Strongly Disagree', 'Moderately Disagree', 'Slightly Disagree', 'Slightly Agree', 'Moderately Agree', 'Strongly Agree']
Happiness	I feel that I am not especially in control of my life.	['Strongly Agree', 'Moderately Agree', 'Slightly Agree', 'Slightly Disagree', 'Moderately Disagree', 'Strongly Disagree']
Happiness	I feel able to take anything on.	['Strongly Disagree', 'Moderately Disagree', 'Slightly Disagree', 'Slightly Agree', 'Moderately Agree', 'Strongly Agree']
Happiness	I feel fully mentally alert.	['Strongly Disagree', 'Moderately Disagree', 'Slightly Disagree', 'Slightly Agree', 'Moderately Agree', 'Strongly Agree']
Happiness	I often experience joy and elation.	['Strongly Disagree', 'Moderately Disagree',

©2017-2018 Arivale Inc. All Rights Reserved. Confidential and Proprietary.

		'Slightly Disagree', 'Slightly Agree', 'Moderately Agree', 'Strongly Agree']
Happiness	I don't find it easy to make decisions.	['Strongly Agree', 'Moderately Agree', 'Slightly Agree', 'Slightly Disagree', 'Moderately Disagree', 'Strongly Disagree']
Happiness	I don't have a particular sense of meaning and purpose in my life.	['Strongly Agree', 'Moderately Agree', 'Slightly Agree', 'Slightly Disagree', 'Moderately Disagree', 'Strongly Disagree']
Happiness	I feel I have a great deal of energy.	['Strongly Disagree', 'Moderately Disagree', 'Slightly Disagree', 'Slightly Agree', 'Moderately Agree', 'Strongly Agree']
Happiness	I usually have a good influence on events.	['Strongly Disagree', 'Moderately Disagree', 'Slightly Disagree', 'Slightly Agree', 'Moderately Agree', 'Strongly Agree']
Happiness	I don't have fun with other people.	['Strongly Agree', 'Moderately Agree', 'Slightly Agree', 'Slightly Disagree', 'Moderately Disagree', 'Strongly Disagree']
Happiness	I don't feel particularly healthy.	['Strongly Agree', 'Moderately Agree', 'Slightly Agree', 'Slightly Disagree', 'Moderately Disagree', 'Strongly Disagree']
Happiness	I don't have particularly happy memories of the past.	['Strongly Agree', 'Moderately Agree', 'Slightly Agree', 'Slightly Disagree', 'Moderately Disagree', 'Strongly Disagree']
Health History	How tall are you?	[None]
Health History	How much do you weigh?	[None]
Health History	Do you currently take prescription medications?	['No', 'Yes']
Health History	Please list all current prescription medications with dosages.	[None]

©2017-2018 Arivale Inc. All Rights Reserved. Confidential and Proprietary.

Health History	Do you currently take over-the-counter medications (e.g. Ibuprofen, Aspirin, Pepto-Bismol)?	['No', 'Rarely/Occasionally', 'Yes']
Health History	Please list all current over-the-counter medications (e.g. Ibuprofen, Aspirin, Pepto-Bismol).	[None]
Health History	Do you currently take supplements (e.g. vitamins, herbs, fish oil, probiotics)?	['No', 'Rarely/Occasionally', 'Yes']
Health History	Please list all current supplements (e.g. vitamins, herbs, fish oil, probiotics) with dosages.	[None]
Health History	Are you currently lactating?	['Yes', 'No']
Health History	Are you adopted?	['Yes', 'No']
Health History	Other Musculoskeletal Injury	['Currently', 'Within the last year', 'More than a year ago', 'Mother', 'Father', 'Sibling(s)', "Mother's side", "Father's side"]
Health History	Fracture	['Currently', 'Within the last year', 'More than a year ago', 'Mother', 'Father', 'Sibling(s)', "Mother's side", "Father's side"]
Health History	Anxiety disorder	['Currently', 'Within the last year', 'More than a year ago', 'Mother', 'Father', 'Sibling(s)', "Mother's side", "Father's side"]
Health History	Schizophrenia	['Currently', 'Within the last year', 'More than a year ago', 'Mother', 'Father', 'Sibling(s)', "Mother's side", "Father's side"]
Health History	Depression	['Currently', 'Within the last year', 'More than a year ago', 'Mother', 'Father', 'Sibling(s)', "Mother's side", "Father's side"]
Health History	Bipolar disorder	['Currently', 'Within the last year', 'More than a year ago', 'Mother', 'Father', 'Sibling(s)',

©2017-2018 Arivale Inc. All Rights Reserved. Confidential and Proprietary.

Arivale
Your scientific path to wellness

		"Mother's side", "Father's side"]
Health History	ADHD or ADD	['Currently', 'Within the last year', 'More than a year ago', 'Mother', 'Father', 'Sibling(s)', "Mother's side", "Father's side"]
Health History	Obsessive-compulsive disorder	['Currently', 'Within the last year', 'More than a year ago', 'Mother', 'Father', 'Sibling(s)', "Mother's side", "Father's side"]
Health History	Anorexia	['Currently', 'Within the last year', 'More than a year ago', 'Mother', 'Father', 'Sibling(s)', "Mother's side", "Father's side"]
Health History	Bulimia	['Currently', 'Within the last year', 'More than a year ago', 'Mother', 'Father', 'Sibling(s)', "Mother's side", "Father's side"]
Health History	Binge eating disorder (BED)	['Currently', 'Within the last year', 'More than a year ago', 'Mother', 'Father', 'Sibling(s)', "Mother's side", "Father's side"]
Health History	Other mental health condition	['Currently', 'Within the last year', 'More than a year ago', 'Mother', 'Father', 'Sibling(s)', "Mother's side", "Father's side"]
Health History	Breast cancer	['Currently', 'Within the last year', 'More than a year ago', 'Mother', 'Father', 'Sibling(s)', "Mother's side", "Father's side"]
Health History	Breast lump (benign)	['Currently', 'Within the last year', 'More than a year ago', 'Mother', 'Father', 'Sibling(s)', "Mother's side", "Father's side"]
Health History	Colon cancer	['Currently', 'Within the last year', 'More than a year ago', 'Mother', 'Father', 'Sibling(s)',

©2017-2018 Arivale Inc. All Rights Reserved. Confidential and Proprietary.

		"Mother's side", "Father's side"]
Health History	Colon polyps (benign)	['Currently', 'Within the last year', 'More than a year ago', 'Mother', 'Father', 'Sibling(s)', "Mother's side", "Father's side"]
Health History	Lung cancer (non-small cell)	['Currently', 'Within the last year', 'More than a year ago', 'Mother', 'Father', 'Sibling(s)', "Mother's side", "Father's side"]
Health History	Lung cancer (small cell)	['Currently', 'Within the last year', 'More than a year ago', 'Mother', 'Father', 'Sibling(s)', "Mother's side", "Father's side"]
Health History	Leukemia	['Currently', 'Within the last year', 'More than a year ago', 'Mother', 'Father', 'Sibling(s)', "Mother's side", "Father's side"]
Health History	Ovarian cancer	['Currently', 'Within the last year', 'More than a year ago', 'Mother', 'Father', 'Sibling(s)', "Mother's side", "Father's side"]
Health History	Endometrial cancer	['Currently', 'Within the last year', 'More than a year ago', 'Mother', 'Father', 'Sibling(s)', "Mother's side", "Father's side"]
Health History	Prostate cancer	['Currently', 'Within the last year', 'More than a year ago', 'Mother', 'Father', 'Sibling(s)', "Mother's side", "Father's side"]
Health History	Liver cancer	['Currently', 'Within the last year', 'More than a year ago', 'Mother', 'Father', 'Sibling(s)', "Mother's side", "Father's side"]
Health History	Kidney cancer	['Currently', 'Within the last year', 'More than a year ago', 'Mother', 'Father', 'Sibling(s)',

©2017-2018 Arivale Inc. All Rights Reserved. Confidential and Proprietary.

		"Mother's side", "Father's side"]
Health History	Cervical cancer	['Currently', 'Within the last year', 'More than a year ago', 'Mother', 'Father', 'Sibling(s)', "Mother's side", "Father's side"]
Health History	Bladder cancer	['Currently', 'Within the last year', 'More than a year ago', 'Mother', 'Father', 'Sibling(s)', "Mother's side", "Father's side"]
Health History	Melanoma	['Currently', 'Within the last year', 'More than a year ago', 'Mother', 'Father', 'Sibling(s)', "Mother's side", "Father's side"]
Health History	Squamous cell carcinoma	['Currently', 'Within the last year', 'More than a year ago', 'Mother', 'Father', 'Sibling(s)', "Mother's side", "Father's side"]
Health History	Basal cell carcinoma	['Currently', 'Within the last year', 'More than a year ago', 'Mother', 'Father', 'Sibling(s)', "Mother's side", "Father's side"]
Health History	Vaginal/vulvar cancer	['Currently', 'Within the last year', 'More than a year ago', 'Mother', 'Father', 'Sibling(s)', "Mother's side", "Father's side"]
Health History	Other cancer	['Currently', 'Within the last year', 'More than a year ago', 'Mother', 'Father', 'Sibling(s)', "Mother's side", "Father's side"]
Health History	Alzheimer's disease or dementia	['Currently', 'Within the last year', 'More than a year ago', 'Mother', 'Father', 'Sibling(s)', "Mother's side", "Father's side"]
Health History	Parkinson's disease	['Currently', 'Within the last year', 'More than a year ago', 'Mother', 'Father', 'Sibling(s)',

©2017-2018 Arivale Inc. All Rights Reserved. Confidential and Proprietary.

		"Mother's side", "Father's side"]
Health History	Essential tremor	['Currently', 'Within the last year', 'More than a year ago', 'Mother', 'Father', 'Sibling(s)', "Mother's side", "Father's side"]
Health History	Mild cognitive impairment (MCI)	['Currently', 'Within the last year', 'More than a year ago', 'Mother', 'Father', 'Sibling(s)', "Mother's side", "Father's side"]
Health History	Multiple sclerosis	['Currently', 'Within the last year', 'More than a year ago', 'Mother', 'Father', 'Sibling(s)', "Mother's side", "Father's side"]
Health History	Other neurocognitive condition	['Currently', 'Within the last year', 'More than a year ago', 'Mother', 'Father', 'Sibling(s)', "Mother's side", "Father's side"]
Health History	Fibroids/leiomyoma	['Currently', 'Within the last year', 'More than a year ago', 'Mother', 'Father', 'Sibling(s)', "Mother's side", "Father's side"]
Health History	Endometriosis	['Currently', 'Within the last year', 'More than a year ago', 'Mother', 'Father', 'Sibling(s)', "Mother's side", "Father's side"]
Health History	Recurrent miscarriage (2+)	['Currently', 'Within the last year', 'More than a year ago', 'Mother', 'Father', 'Sibling(s)', "Mother's side", "Father's side"]
Health History	Pelvic inflammatory disease	['Currently', 'Within the last year', 'More than a year ago', 'Mother', 'Father', 'Sibling(s)', "Mother's side", "Father's side"]
Health History	Excessive menstruation	['Currently', 'Within the last year', 'More than a year ago', 'Mother', 'Father', 'Sibling(s)',

©2017-2018 Arivale Inc. All Rights Reserved. Confidential and Proprietary.

		"Mother's side", "Father's side"]
Health History	Hysterectomy/oophorectomy	['Currently', 'Within the last year', 'More than a year ago', 'Mother', 'Father', 'Sibling(s)', "Mother's side", "Father's side"]
Health History	Polycystic ovarian syndrome	['Currently', 'Within the last year', 'More than a year ago', 'Mother', 'Father', 'Sibling(s)', "Mother's side", "Father's side"]
Health History	HIV	['Currently', 'Within the last year', 'More than a year ago', 'Mother', 'Father', 'Sibling(s)', "Mother's side", "Father's side"]
Health History	Shingles	['Currently', 'Within the last year', 'More than a year ago', 'Mother', 'Father', 'Sibling(s)', "Mother's side", "Father's side"]
Health History	HPV	['Currently', 'Within the last year', 'More than a year ago', 'Mother', 'Father', 'Sibling(s)', "Mother's side", "Father's side"]
Health History	Herpes type 1 or 2 (HSV)	['Currently', 'Within the last year', 'More than a year ago', 'Mother', 'Father', 'Sibling(s)', "Mother's side", "Father's side"]
Health History	Cytomegalovirus (CMV)	['Currently', 'Within the last year', 'More than a year ago', 'Mother', 'Father', 'Sibling(s)', "Mother's side", "Father's side"]
Health History	Epstein-Barr virus (EBV)	['Currently', 'Within the last year', 'More than a year ago', 'Mother', 'Father', 'Sibling(s)', "Mother's side", "Father's side"]
Health History	Lyme disease	['Currently', 'Within the last year', 'More than a year ago', 'Mother', 'Father', 'Sibling(s)',

©2017-2018 Arivale Inc. All Rights Reserved. Confidential and Proprietary.

Arivale
Your scientific path to wellness

		"Mother's side", "Father's side"]
Health History	Other infectious disease	['Currently', 'Within the last year', 'More than a year ago', 'Mother', 'Father', 'Sibling(s)', "Mother's side", "Father's side"]
Health History	Gastroesophageal reflux disease (GERD) or Barret's esophagus	['Currently', 'Within the last year', 'More than a year ago', 'Mother', 'Father', 'Sibling(s)', "Mother's side", "Father's side"]
Health History	Peptic ulcer	['Currently', 'Within the last year', 'More than a year ago', 'Mother', 'Father', 'Sibling(s)', "Mother's side", "Father's side"]
Health History	Gastritis	['Currently', 'Within the last year', 'More than a year ago', 'Mother', 'Father', 'Sibling(s)', "Mother's side", "Father's side"]
Health History	Crohn's disease (other or unknown type)	['Currently', 'Within the last year', 'More than a year ago', 'Mother', 'Father', 'Sibling(s)', "Mother's side", "Father's side"]
Health History	Ulcerative colitis	['Currently', 'Within the last year', 'More than a year ago', 'Mother', 'Father', 'Sibling(s)', "Mother's side", "Father's side"]
Health History	Diverticulosis	['Currently', 'Within the last year', 'More than a year ago', 'Mother', 'Father', 'Sibling(s)', "Mother's side", "Father's side"]
Health History	Celiac disease	['Currently', 'Within the last year', 'More than a year ago', 'Mother', 'Father', 'Sibling(s)', "Mother's side", "Father's side"]
Health History	Irritable bowel syndrome (IBS)	['Currently', 'Within the last year', 'More than a year ago', 'Mother', 'Father', 'Sibling(s)',

©2017-2018 Arivale Inc. All Rights Reserved. Confidential and Proprietary.

		"Mother's side", "Father's side"]
Health History	Other gastrointestinal condition	['Currently', 'Within the last year', 'More than a year ago', 'Mother', 'Father', 'Sibling(s)', "Mother's side", "Father's side"]
Health History	Hepatitis C	['Currently', 'Within the last year', 'More than a year ago', 'Mother', 'Father', 'Sibling(s)', "Mother's side", "Father's side"]
Health History	Other hepatitis (A, B, D, E or autoimmune)	['Currently', 'Within the last year', 'More than a year ago', 'Mother', 'Father', 'Sibling(s)', "Mother's side", "Father's side"]
Health History	Fatty liver disease (NAFLD or NASH)	['Currently', 'Within the last year', 'More than a year ago', 'Mother', 'Father', 'Sibling(s)', "Mother's side", "Father's side"]
Health History	Cirrhosis	['Currently', 'Within the last year', 'More than a year ago', 'Mother', 'Father', 'Sibling(s)', "Mother's side", "Father's side"]
Health History	Liver failure	['Currently', 'Within the last year', 'More than a year ago', 'Mother', 'Father', 'Sibling(s)', "Mother's side", "Father's side"]
Health History	Liver abscess or cyst	['Currently', 'Within the last year', 'More than a year ago', 'Mother', 'Father', 'Sibling(s)', "Mother's side", "Father's side"]
Health History	Pancreatitis	['Currently', 'Within the last year', 'More than a year ago', 'Mother', 'Father', 'Sibling(s)', "Mother's side", "Father's side"]
Health History	Gallstones	['Currently', 'Within the last year', 'More than a year ago', 'Mother', 'Father', 'Sibling(s)',

©2017-2018 Arivale Inc. All Rights Reserved. Confidential and Proprietary.

Arivale

Your scientific path to wellness

		"Mother's side", "Father's side"]
Health History	Other liver, pancreas, or gallbladder condition	['Currently', 'Within the last year', 'More than a year ago', 'Mother', 'Father', 'Sibling(s)', "Mother's side", "Father's side"]
Health History	Kidney stones	['Currently', 'Within the last year', 'More than a year ago', 'Mother', 'Father', 'Sibling(s)', "Mother's side", "Father's side"]
Health History	Kidney disease	['Currently', 'Within the last year', 'More than a year ago', 'Mother', 'Father', 'Sibling(s)', "Mother's side", "Father's side"]
Health History	Kidney failure	['Currently', 'Within the last year', 'More than a year ago', 'Mother', 'Father', 'Sibling(s)', "Mother's side", "Father's side"]
Health History	Bladder infection	['Currently', 'Within the last year', 'More than a year ago', 'Mother', 'Father', 'Sibling(s)', "Mother's side", "Father's side"]
Health History	Urinary incontinence	['Currently', 'Within the last year', 'More than a year ago', 'Mother', 'Father', 'Sibling(s)', "Mother's side", "Father's side"]
Health History	Hypertension (high blood pressure)	['Currently', 'Within the last year', 'More than a year ago', 'Mother', 'Father', 'Sibling(s)', "Mother's side", "Father's side"]
Health History	Elevated cholesterol or triglycerides	['Currently', 'Within the last year', 'More than a year ago', 'Mother', 'Father', 'Sibling(s)', "Mother's side", "Father's side"]
Health History	Coronary artery disease	['Currently', 'Within the last year', 'More than a year ago', 'Mother', 'Father', 'Sibling(s)',

©2017-2018 Arivale Inc. All Rights Reserved. Confidential and Proprietary.

		"Mother's side", "Father's side"]
Health History	Heart attack (myocardial infarction)	['Currently', 'Within the last year', 'More than a year ago', 'Mother', 'Father', 'Sibling(s)', "Mother's side", "Father's side"]
Health History	Abnormal heart rhythm (arrhythmia)	['Currently', 'Within the last year', 'More than a year ago', 'Mother', 'Father', 'Sibling(s)', "Mother's side", "Father's side"]
Health History	Heart failure	['Currently', 'Within the last year', 'More than a year ago', 'Mother', 'Father', 'Sibling(s)', "Mother's side", "Father's side"]
Health History	Heart valve disease	['Currently', 'Within the last year', 'More than a year ago', 'Mother', 'Father', 'Sibling(s)', "Mother's side", "Father's side"]
Health History	Congenital heart disease	['Currently', 'Within the last year', 'More than a year ago', 'Mother', 'Father', 'Sibling(s)', "Mother's side", "Father's side"]
Health History	Heart muscle disease (cardiomyopathy)	['Currently', 'Within the last year', 'More than a year ago', 'Mother', 'Father', 'Sibling(s)', "Mother's side", "Father's side"]
Health History	Pericardial disease	['Currently', 'Within the last year', 'More than a year ago', 'Mother', 'Father', 'Sibling(s)', "Mother's side", "Father's side"]
Health History	Aortic disease	['Currently', 'Within the last year', 'More than a year ago', 'Mother', 'Father', 'Sibling(s)', "Mother's side", "Father's side"]
Health History	Marfan syndrome	['Currently', 'Within the last year', 'More than a year ago', 'Mother', 'Father', 'Sibling(s)',

©2017-2018 Arivale Inc. All Rights Reserved. Confidential and Proprietary.

		"Mother's side", "Father's side"]
Health History	Vascular disease (blood vessel disease)	['Currently', 'Within the last year', 'More than a year ago', 'Mother', 'Father', 'Sibling(s)', "Mother's side", "Father's side"]
Health History	Blood clots	['Currently', 'Within the last year', 'More than a year ago', 'Mother', 'Father', 'Sibling(s)', "Mother's side", "Father's side"]
Health History	Stroke (other or unknown type)	['Currently', 'Within the last year', 'More than a year ago', 'Mother', 'Father', 'Sibling(s)', "Mother's side", "Father's side"]
Health History	Stroke (ischemic)	['Currently', 'Within the last year', 'More than a year ago', 'Mother', 'Father', 'Sibling(s)', "Mother's side", "Father's side"]
Health History	Stroke (hemorrhagic)	['Currently', 'Within the last year', 'More than a year ago', 'Mother', 'Father', 'Sibling(s)', "Mother's side", "Father's side"]
Health History	Transient ischemic attack	['Currently', 'Within the last year', 'More than a year ago', 'Mother', 'Father', 'Sibling(s)', "Mother's side", "Father's side"]
Health History	Psoriasis	['Currently', 'Within the last year', 'More than a year ago', 'Mother', 'Father', 'Sibling(s)', "Mother's side", "Father's side"]
Health History	Eczema	['Currently', 'Within the last year', 'More than a year ago', 'Mother', 'Father', 'Sibling(s)', "Mother's side", "Father's side"]
Health History	Acne	['Currently', 'Within the last year', 'More than a year ago', 'Mother', 'Father', 'Sibling(s)',

©2017-2018 Arivale Inc. All Rights Reserved. Confidential and Proprietary.

		"Mother's side", "Father's side"]
Health History	Rosacea	['Currently', 'Within the last year', 'More than a year ago', 'Mother', 'Father', 'Sibling(s)', "Mother's side", "Father's side"]
Health History	Vitiligo	['Currently', 'Within the last year', 'More than a year ago', 'Mother', 'Father', 'Sibling(s)', "Mother's side", "Father's side"]
Health History	Warts	['Currently', 'Within the last year', 'More than a year ago', 'Mother', 'Father', 'Sibling(s)', "Mother's side", "Father's side"]
Health History	Cellulitis	['Currently', 'Within the last year', 'More than a year ago', 'Mother', 'Father', 'Sibling(s)', "Mother's side", "Father's side"]
Health History	Actinic keratosis	['Currently', 'Within the last year', 'More than a year ago', 'Mother', 'Father', 'Sibling(s)', "Mother's side", "Father's side"]
Health History	Mouth ulcers	['Currently', 'Within the last year', 'More than a year ago', 'Mother', 'Father', 'Sibling(s)', "Mother's side", "Father's side"]
Health History	Chronic sinus infections	['Currently', 'Within the last year', 'More than a year ago', 'Mother', 'Father', 'Sibling(s)', "Mother's side", "Father's side"]
Health History	Inner ear disorder (infection, vertigo, Meniere's)	['Currently', 'Within the last year', 'More than a year ago', 'Mother', 'Father', 'Sibling(s)', "Mother's side", "Father's side"]
Health History	Throat condition (chronic strep, irritation, chronic clearing of throat)	['Currently', 'Within the last year', 'More than a year ago', 'Mother', 'Father', 'Sibling(s)',

©2017-2018 Arivale Inc. All Rights Reserved. Confidential and Proprietary.

		"Mother's side", "Father's side"]
Health History	Head or neck masses	['Currently', 'Within the last year', 'More than a year ago', 'Mother', 'Father', 'Sibling(s)', "Mother's side", "Father's side"]
Health History	Glaucoma	['Currently', 'Within the last year', 'More than a year ago', 'Mother', 'Father', 'Sibling(s)', "Mother's side", "Father's side"]
Health History	Other skin conditions	['Currently', 'Within the last year', 'More than a year ago', 'Mother', 'Father', 'Sibling(s)', "Mother's side", "Father's side"]
Health History	Other cardiovascular condition	['Currently', 'Within the last year', 'More than a year ago', 'Mother', 'Father', 'Sibling(s)', "Mother's side", "Father's side"]
Health History	Other bladder or kidney condition	['Currently', 'Within the last year', 'More than a year ago', 'Mother', 'Father', 'Sibling(s)', "Mother's side", "Father's side"]
Health History	Cataracts	['Currently', 'Within the last year', 'More than a year ago', 'Mother', 'Father', 'Sibling(s)', "Mother's side", "Father's side"]
Health History	Macular degeneration	['Currently', 'Within the last year', 'More than a year ago', 'Mother', 'Father', 'Sibling(s)', "Mother's side", "Father's side"]
Health History	Other eye, ear, nose, or throat condition	['Currently', 'Within the last year', 'More than a year ago', 'Mother', 'Father', 'Sibling(s)', "Mother's side", "Father's side"]
Health History	Emphysema	['Currently', 'Within the last year', 'More than a year ago', 'Mother', 'Father', 'Sibling(s)',

©2017-2018 Arivale Inc. All Rights Reserved. Confidential and Proprietary.

		"Mother's side", "Father's side"]
Health History	Shortness of breath	['Currently', 'Within the last year', 'More than a year ago', 'Mother', 'Father', 'Sibling(s)', "Mother's side", "Father's side"]
Health History	Asthma	['Currently', 'Within the last year', 'More than a year ago', 'Mother', 'Father', 'Sibling(s)', "Mother's side", "Father's side"]
Health History	Chronic obstructive pulmonary disease (COPD)	['Currently', 'Within the last year', 'More than a year ago', 'Mother', 'Father', 'Sibling(s)', "Mother's side", "Father's side"]
Health History	Bronchitis (chronic)	['Currently', 'Within the last year', 'More than a year ago', 'Mother', 'Father', 'Sibling(s)', "Mother's side", "Father's side"]
Health History	Pulmonary hypertension (high blood pressure in the lungs)	['Currently', 'Within the last year', 'More than a year ago', 'Mother', 'Father', 'Sibling(s)', "Mother's side", "Father's side"]
Health History	Sarcoidosis	['Currently', 'Within the last year', 'More than a year ago', 'Mother', 'Father', 'Sibling(s)', "Mother's side", "Father's side"]
Health History	Collapse of part or all of the lung (atelectasis or pneumothorax)	['Currently', 'Within the last year', 'More than a year ago', 'Mother', 'Father', 'Sibling(s)', "Mother's side", "Father's side"]
Health History	Lung infection (e.g. pneumonia)	['Currently', 'Within the last year', 'More than a year ago', 'Mother', 'Father', 'Sibling(s)', "Mother's side", "Father's side"]
Health History	Blocked lung artery (pulmonary embolus)	['Currently', 'Within the last year', 'More than a year ago', 'Mother', 'Father', 'Sibling(s)',

©2017-2018 Arivale Inc. All Rights Reserved. Confidential and Proprietary.

		"Mother's side", "Father's side"]
Health History	Insomnia	['Currently', 'Within the last year', 'More than a year ago', 'Mother', 'Father', 'Sibling(s)', "Mother's side", "Father's side"]
Health History	Sleep apnea	['Currently', 'Within the last year', 'More than a year ago', 'Mother', 'Father', 'Sibling(s)', "Mother's side", "Father's side"]
Health History	Restless leg syndrome	['Currently', 'Within the last year', 'More than a year ago', 'Mother', 'Father', 'Sibling(s)', "Mother's side", "Father's side"]
Health History	Narcolepsy	['Currently', 'Within the last year', 'More than a year ago', 'Mother', 'Father', 'Sibling(s)', "Mother's side", "Father's side"]
Health History	Other lung condition	['Currently', 'Within the last year', 'More than a year ago', 'Mother', 'Father', 'Sibling(s)', "Mother's side", "Father's side"]
Health History	Other sleep condition	['Currently', 'Within the last year', 'More than a year ago', 'Mother', 'Father', 'Sibling(s)', "Mother's side", "Father's side"]
Health History	Multiple sclerosis	['Currently', 'Within the last year', 'More than a year ago', 'Mother', 'Father', 'Sibling(s)', "Mother's side", "Father's side"]
Health History	Rheumatoid arthritis	['Currently', 'Within the last year', 'More than a year ago', 'Mother', 'Father', 'Sibling(s)', "Mother's side", "Father's side"]
Health History	Sjogren's syndrome	['Currently', 'Within the last year', 'More than a year ago', 'Mother', 'Father', 'Sibling(s)',

©2017-2018 Arivale Inc. All Rights Reserved. Confidential and Proprietary.

		"Mother's side", "Father's side"]
Health History	Lupus	['Currently', 'Within the last year', 'More than a year ago', 'Mother', 'Father', 'Sibling(s)', "Mother's side", "Father's side"]
Health History	Myasthenia Gravis	['Currently', 'Within the last year', 'More than a year ago', 'Mother', 'Father', 'Sibling(s)', "Mother's side", "Father's side"]
Health History	Type 1 diabetes	['Currently', 'Within the last year', 'More than a year ago', 'Mother', 'Father', 'Sibling(s)', "Mother's side", "Father's side"]
Health History	Other autoimmune conditions	['Currently', 'Within the last year', 'More than a year ago', 'Mother', 'Father', 'Sibling(s)', "Mother's side", "Father's side"]
Health History	Periodontal disease	['Currently', 'Within the last year', 'More than a year ago', 'Mother', 'Father', 'Sibling(s)', "Mother's side", "Father's side"]
Health History	Other oral health conditions	['Currently', 'Within the last year', 'More than a year ago', 'Mother', 'Father', 'Sibling(s)', "Mother's side", "Father's side"]
Health History	BRCA1 associated hereditary breast cancer	['Currently', 'Within the last year', 'More than a year ago', 'Mother', 'Father', 'Sibling(s)', "Mother's side", "Father's side"]
Health History	BRCA2 associated hereditary breast cancer	['Currently', 'Within the last year', 'More than a year ago', 'Mother', 'Father', 'Sibling(s)', "Mother's side", "Father's side"]
Health History	BRCA1 associated hereditary ovarian cancer	['Currently', 'Within the last year', 'More than a year ago', 'Mother', 'Father', 'Sibling(s)',

©2017-2018 Arivale Inc. All Rights Reserved. Confidential and Proprietary.

		"Mother's side", "Father's side"]
Health History	BRCA2 associated hereditary ovarian cancer	['Currently', 'Within the last year', 'More than a year ago', 'Mother', 'Father', 'Sibling(s)', "Mother's side", "Father's side"]
Health History	Lynch syndrome	['Currently', 'Within the last year', 'More than a year ago', 'Mother', 'Father', 'Sibling(s)', "Mother's side", "Father's side"]
Health History	Brugada syndrome	['Currently', 'Within the last year', 'More than a year ago', 'Mother', 'Father', 'Sibling(s)', "Mother's side", "Father's side"]
Health History	Li Fraumeni syndrome	['Currently', 'Within the last year', 'More than a year ago', 'Mother', 'Father', 'Sibling(s)', "Mother's side", "Father's side"]
Health History	Malignant hyperthermia	['Currently', 'Within the last year', 'More than a year ago', 'Mother', 'Father', 'Sibling(s)', "Mother's side", "Father's side"]
Health History	Hyperthyroidism (overactive thyroid)	['Currently', 'Within the last year', 'More than a year ago', 'Mother', 'Father', 'Sibling(s)', "Mother's side", "Father's side"]
Health History	Hypothyroidism (underactive thyroid)	['Currently', 'Within the last year', 'More than a year ago', 'Mother', 'Father', 'Sibling(s)', "Mother's side", "Father's side"]
Health History	Thyroid nodule	['Currently', 'Within the last year', 'More than a year ago', 'Mother', 'Father', 'Sibling(s)', "Mother's side", "Father's side"]
Health History	Hashimoto's disease	['Currently', 'Within the last year', 'More than a year ago', 'Mother', 'Father', 'Sibling(s)',

©2017-2018 Arivale Inc. All Rights Reserved. Confidential and Proprietary.

		"Mother's side", "Father's side"]
Health History	Graves' disease	['Currently', 'Within the last year', 'More than a year ago', 'Mother', 'Father', 'Sibling(s)', "Mother's side", "Father's side"]
Health History	Irregular menstrual bleeding (not due to pre-menopause/menopause)	['Currently', 'Within the last year', 'More than a year ago', 'Mother', 'Father', 'Sibling(s)', "Mother's side", "Father's side"]
Health History	No menstrual bleeding (amenorrhea, not due to pre-menopause/menopause)	['Currently', 'Within the last year', 'More than a year ago', 'Mother', 'Father', 'Sibling(s)', "Mother's side", "Father's side"]
Health History	Prostate enlargement or benign prostatic hyperplasia	['Currently', 'Within the last year', 'More than a year ago', 'Mother', 'Father', 'Sibling(s)', "Mother's side", "Father's side"]
Health History	Prostatitis (prostate inflammation)	['Currently', 'Within the last year', 'More than a year ago', 'Mother', 'Father', 'Sibling(s)', "Mother's side", "Father's side"]
Health History	Prostate nodules	['Currently', 'Within the last year', 'More than a year ago', 'Mother', 'Father', 'Sibling(s)', "Mother's side", "Father's side"]
Health History	Erectile dysfunction	['Currently', 'Within the last year', 'More than a year ago', 'Mother', 'Father', 'Sibling(s)', "Mother's side", "Father's side"]
Health History	Male infertility	['Currently', 'Within the last year', 'More than a year ago', 'Mother', 'Father', 'Sibling(s)', "Mother's side", "Father's side"]
Health History	Facial paralysis	['Currently', 'Within the last year', 'More than a year ago', 'Mother', 'Father', 'Sibling(s)',

©2017-2018 Arivale Inc. All Rights Reserved. Confidential and Proprietary.

		"Mother's side", "Father's side"]
Health History	Lactose intolerance	['Currently', 'Within the last year', 'More than a year ago', 'Mother', 'Father', 'Sibling(s)', "Mother's side", "Father's side"]
Health History	Seasonal allergies	['Currently', 'Within the last year', 'More than a year ago', 'Mother', 'Father', 'Sibling(s)', "Mother's side", "Father's side"]
Health History	Other allergies affecting either the eyes, ears, nose, or throat (e.g. dust, mold, or pet allergies)	['Currently', 'Within the last year', 'More than a year ago', 'Mother', 'Father', 'Sibling(s)', "Mother's side", "Father's side"]
Health History	Other heritable (Mendelian) disorder	['Currently', 'Within the last year', 'More than a year ago', 'Mother', 'Father', 'Sibling(s)', "Mother's side", "Father's side"]
Health History	Migraines	['Currently', 'Within the last year', 'More than a year ago', 'Mother', 'Father', 'Sibling(s)', "Mother's side", "Father's side"]
Health History	Post-traumatic stress disorder (PTSD)	['Currently', 'Within the last year', 'More than a year ago', 'Mother', 'Father', 'Sibling(s)', "Mother's side", "Father's side"]
Health History	Seizures or Epilepsy	['Currently', 'Within the last year', 'More than a year ago', 'Mother', 'Father', 'Sibling(s)', "Mother's side", "Father's side"]
Health History	Aneurysm	['Currently', 'Within the last year', 'More than a year ago', 'Mother', 'Father', 'Sibling(s)', "Mother's side", "Father's side"]
Health History	Heart murmur	['Currently', 'Within the last year', 'More than a year ago', 'Mother', 'Father', 'Sibling(s)',

©2017-2018 Arivale Inc. All Rights Reserved. Confidential and Proprietary.

		"Mother's side", "Father's side"]
Health History	Concussion or traumatic brain injury	['Currently', 'Within the last year', 'More than a year ago', 'Mother', 'Father', 'Sibling(s)', "Mother's side", "Father's side"]
Health History	Alcohol abuse	['Currently', 'Within the last year', 'More than a year ago', 'Mother', 'Father', 'Sibling(s)', "Mother's side", "Father's side"]
Health History	Drug abuse	['Currently', 'Within the last year', 'More than a year ago', 'Mother', 'Father', 'Sibling(s)', "Mother's side", "Father's side"]
Health History	Type 2 diabetes	['Currently', 'Within the last year', 'More than a year ago', 'Mother', 'Father', 'Sibling(s)', "Mother's side", "Father's side"]
Health History	Gout	['Currently', 'Within the last year', 'More than a year ago', 'Mother', 'Father', 'Sibling(s)', "Mother's side", "Father's side"]
Health History	Gestational diabetes	['Currently', 'Within the last year', 'More than a year ago', 'Mother', 'Father', 'Sibling(s)', "Mother's side", "Father's side"]
Health History	Metabolic syndrome	['Currently', 'Within the last year', 'More than a year ago', 'Mother', 'Father', 'Sibling(s)', "Mother's side", "Father's side"]
Health History	Other metabolic condition	['Currently', 'Within the last year', 'More than a year ago', 'Mother', 'Father', 'Sibling(s)', "Mother's side", "Father's side"]
Health History	Anemia (iron deficiency)	['Currently', 'Within the last year', 'More than a year ago', 'Mother', 'Father', 'Sibling(s)',

©2017-2018 Arivale Inc. All Rights Reserved. Confidential and Proprietary.

		"Mother's side", "Father's side"]
Health History	Anemia (pernicious or vitamin deficiency anemia)	['Currently', 'Within the last year', 'More than a year ago', 'Mother', 'Father', 'Sibling(s)', "Mother's side", "Father's side"]
Health History	Anemia (other or unknown type)	['Currently', 'Within the last year', 'More than a year ago', 'Mother', 'Father', 'Sibling(s)', "Mother's side", "Father's side"]
Health History	Chronic Fatigue Syndrome	['Currently', 'Within the last year', 'More than a year ago', 'Mother', 'Father', 'Sibling(s)', "Mother's side", "Father's side"]
Health History	Fibromyalgia	['Currently', 'Within the last year', 'More than a year ago', 'Mother', 'Father', 'Sibling(s)', "Mother's side", "Father's side"]
Health History	Other conditions involving chronic fatigue	['Currently', 'Within the last year', 'More than a year ago', 'Mother', 'Father', 'Sibling(s)', "Mother's side", "Father's side"]
Health History	Colonoscopy	['Within the last 3 months', 'Within the last year', 'More than a year ago']
Health History	Mammogram	['Within the last 3 months', 'Within the last year', 'More than a year ago']
Health History	Prostate exam	['Within the last 3 months', 'Within the last year', 'More than a year ago']
Health History	Bone density scan (e.g. DEXA)	['Within the last 3 months', 'Within the last year', 'More than a year ago']
Health History	Other screening exam	['Within the last 3 months', 'Within the last year', 'More than a year ago']
Health History	Chemotherapy	['Within the last 3 months', 'Within the last year', 'More than a year ago']

©2017-2018 Arivale Inc. All Rights Reserved. Confidential and Proprietary.

Health History	Radiation therapy	['Within the last 3 months', 'Within the last year', 'More than a year ago']
Health History	Immunotherapy	['Within the last 3 months', 'Within the last year', 'More than a year ago']
Health History	Blood transfusion	['Within the last 3 months', 'Within the last year', 'More than a year ago']
Health History	Other medical treatments	['Within the last 3 months', 'Within the last year', 'More than a year ago']
Health History	Liver transplant	['Within the last 3 months', 'Within the last year', 'More than a year ago']
Health History	Kidney transplant	['Within the last 3 months', 'Within the last year', 'More than a year ago']
Health History	Bone marrow transplant	['Within the last 3 months', 'Within the last year', 'More than a year ago']
Health History	Heart transplant	['Within the last 3 months', 'Within the last year', 'More than a year ago']
Health History	Other organ transplant	['Within the last 3 months', 'Within the last year', 'More than a year ago']
Health History	Bariatric (weight loss) surgery	['Within the last 3 months', 'Within the last year', 'More than a year ago']
Health History	Surgery for cancer (e.g. tumor removal)	['Within the last 3 months', 'Within the last year', 'More than a year ago']
Health History	Gallbladder removal surgery	['Within the last 3 months', 'Within the last year', 'More than a year ago']
Health History	Sinus or septum surgery	['Within the last 3 months', 'Within the last year', 'More than a year ago']
Health History	Cardiac catheterization	['Within the last 3 months', 'Within the last year', 'More than a year ago']
Health History	Pacemaker or implantable cardiac defibrillator	['Within the last 3 months', 'Within the last year', 'More than a year ago']

©2017-2018 Arivale Inc. All Rights Reserved. Confidential and Proprietary.

Health History	Appendix removal	['Within the last 3 months', 'Within the last year', 'More than a year ago']
Health History	Other surgery or major medical procedure	['Within the last 3 months', 'Within the last year', 'More than a year ago']
Health History	Flu vaccine	['Within the last 3 months', 'Within the last year', 'More than a year ago']
Health History	Pneumonia vaccine	['Within the last 3 months', 'Within the last year', 'More than a year ago']
Health History	Diphtheria, Tetanus, or Pertussis vaccine	['Within the last 3 months', 'Within the last year', 'More than a year ago']
Health History	Shingles vaccine	['Within the last 3 months', 'Within the last year', 'More than a year ago']
Health History	Other vaccine	['Within the last 3 months', 'Within the last year', 'More than a year ago']
Health History	Cholesterol-lowering medications	['Currently', 'Formerly']
Health History	Blood sugar lowering medications	['Currently', 'Formerly']
Health History	Blood pressure lowering medications	['Currently', 'Formerly']
Health History	Psychiatric medications (e.g. antidepressants, anti-anxiety medications, stimulants)	['Currently', 'Formerly']
Health History	Anti-coagulation medications (e.g. blood thinners)	['Currently', 'Formerly']
Health History	Osteoporosis medications	['Currently', 'Formerly']
Health History	Asthma medications	['Currently', 'Formerly']
Health History	Allergy medications	['Currently', 'Formerly']
Health History	Are you post-menopausal?	['No', 'Yes']
Health History	Do you have a primary care provider (e.g. family physician, internist, naturopathic physician)?	['Yes', 'No']
Health History	Are you under the care of one or more of the following specialists?	['Cardiologist', 'Endocrinologist', 'OB-GYN', 'Oncologist', 'Gastroenterologist', 'Other']

©2017-2018 Arivale Inc. All Rights Reserved. Confidential and Proprietary.

Health History	Do you have regular dental exams?	['Yes', 'No']
Health History	Huntington's disease	['Currently', 'Within the last year', 'More than a year ago', 'Mother', 'Father', 'Sibling(s)', "Mother's side", "Father's side"]
Health History	Other gynecological conditions	['Currently', 'Within the last year', 'More than a year ago', 'Mother', 'Father', 'Sibling(s)', "Mother's side", "Father's side"]
Health History	Antibiotics	['Within the last 3 months', 'Within the last year', 'More than a year ago']
Health History	Age at the time of menopause (age at final menstrual period)	[None]
Health History	When was your last (most recent) menstrual period?	[None]
Health History	Other eating disorder	['Currently', 'Within the last year', 'More than a year ago', 'Mother', 'Father', 'Sibling(s)', "Mother's side", "Father's side"]
Health History	What is your biological sex?	['Male', 'Female']
Health History	Non-celiac gluten sensitivity	['Currently', 'Within the last year', 'More than a year ago', 'Mother', 'Father', 'Sibling(s)', "Mother's side", "Father's side"]
Health History	Polycystic kidney disease	['Currently', 'Within the last year', 'More than a year ago', 'Mother', 'Father', 'Sibling(s)', "Mother's side", "Father's side"]
Health History	Crohn's disease (ileal)	['Currently', 'Within the last year', 'More than a year ago', 'Mother', 'Father', 'Sibling(s)', "Mother's side", "Father's side"]
Health History	Crohn's disease (colonic)	['Currently', 'Within the last year', 'More than a year ago', 'Mother', 'Father', 'Sibling(s)', "Mother's side", "Father's side"]

©2017-2018 Arivale Inc. All Rights Reserved. Confidential and Proprietary.

Health History	Alopecia (hair loss)	['Currently', 'Within the last year', 'More than a year ago', 'Mother', 'Father', 'Sibling(s)', "Mother's side", "Father's side"]
Health History	Cystic fibrosis	['Currently', 'Within the last year', 'More than a year ago', 'Mother', 'Father', 'Sibling(s)', "Mother's side", "Father's side"]
Health History	Pap smear	['Within the last 3 months', 'Within the last year', 'More than a year ago']
Health History	Routine blood donations	['Within the last 3 months', 'Within the last year', 'More than a year ago']
Health History	Do you have any conditions not previously listed? If so, please list them here.	[None]
Health History	Do you wear dentures?	['Yes', 'No']
Health History	Back pain or back problems	['Currently', 'Within the last year', 'More than a year ago', 'Mother', 'Father', 'Sibling(s)', "Mother's side", "Father's side"]
Health History	Osteoarthritis	['Currently', 'Within the last year', 'More than a year ago', 'Mother', 'Father', 'Sibling(s)', "Mother's side", "Father's side"]
Health History	Tendinopathy (e.g. achilles tendinopathy)	['Currently', 'Within the last year', 'More than a year ago', 'Mother', 'Father', 'Sibling(s)', "Mother's side", "Father's side"]
Health History	Ligament injuries (e.g. ACL tear or rupture)	['Currently', 'Within the last year', 'More than a year ago', 'Mother', 'Father', 'Sibling(s)', "Mother's side", "Father's side"]
Health History	Carpal tunnel syndrome	['Currently', 'Within the last year', 'More than a year ago', 'Mother', 'Father', 'Sibling(s)', "Mother's side", "Father's side"]

©2017-2018 Arivale Inc. All Rights Reserved. Confidential and Proprietary.

Health History	Osteoporosis	['Currently', 'Within the last year', 'More than a year ago', 'Mother', 'Father', 'Sibling(s)', "Mother's side", "Father's side"]
Journey	Overall, I feel my health is on the right track.	['Strongly disagree', 'Disagree', 'Neither Disagree Nor Agree', 'Agree', 'Strongly agree']
Journey	I feel empowered to do the things I love in life.	['Strongly disagree', 'Disagree', 'Neither Disagree Nor Agree', 'Agree', 'Strongly agree']
Journey	I feel rundown and don't have much energy.	['Strongly disagree', 'Disagree', 'Neither Disagree Nor Agree', 'Agree', 'Strongly agree']
Journey	I feel like I've lost control of my life.	['Strongly disagree', 'Disagree', 'Neither Disagree Nor Agree', 'Agree', 'Strongly agree']
Journey	Lately, I'm unable to concentrate, I lose focus.	['Strongly disagree', 'Disagree', 'Neither Disagree Nor Agree', 'Agree', 'Strongly agree']
Journey	I'm satisfied with the way I'm living my life.	['Strongly disagree', 'Disagree', 'Neither Disagree Nor Agree', 'Agree', 'Strongly agree']
Journey	I know how to live a healthy life.	['Strongly disagree', 'Disagree', 'Neither Disagree Nor Agree', 'Agree', 'Strongly agree']
Journey	What do you hope to achieve on your wellness journey?	[None]
Journey	If you had an extra hour each day, how would you spend it?	[None]
Journey	What's the greatest source of stress in your life right now?	[None]
Journey	What do you look forward to most each day?	[None]
Journey	Please select the goal which is most important for you as you start your Arivale journey.	['Improve fitness', 'Lose weight / improve body composition', 'Reduce stress', 'Improve diet / nutrition', 'Improve digestion / gut health', 'Improve sleep', 'Other']
Journey	If you choose "other", please specify the goal that is most important to you as you start your Arivale journey.	[None]

©2017-2018 Arivale Inc. All Rights Reserved. Confidential and Proprietary.

Journey	How would you rate your quality of life?	['Very poor', 'Poor', 'Neither poor nor good', 'Good', 'Very good']
Journey	To what extent do you feel that physical pain prevents you from doing what you need to do?	['Not at all', 'A little', 'A moderate amount', 'Very much', 'An extreme amount']
Journey	I prefer my Arivale Coach to be:	["Fast tempo. Direct. To the point. I want to get to the point quickly; don't beat around the bush.", "Measured tempo. Conversational. I want time to absorb the information; I don't want to feel rushed; I like to talk through things."]
Journey	When I'm focused on making changes in my life I:	['Am all in. I like to do everything all at once. Lifestyle overhaul works best for me.', 'Walk before I run. I make one or two changes at a time. I like to master a change for a while before I move on.', "Take baby steps. I don't have time or bandwidth for anything but the smallest changes.", "I'm not interested in making changes. I'm here for the information."]
Journey	When it comes to accountability:	['Be strict. I need you to hold me accountable no matter what. Ask me the hard questions even when it might make me uncomfortable.', "Let's collaborate. I want to talk about what system of accountability might work for me. Let's figure this out together.", "I need a cheerleader. Please just encourage me. I'm doing the best I can right now, and I need your unwavering support.", "I'm self-directed. I'm going to work on these changes on my own and I'll

©2017-2018 Arivale Inc. All Rights Reserved. Confidential and Proprietary.

		report back when we talk next."]
Journey	When focusing on making a behavior change, I want my Arivale Coach to:	["Tell me exactly what I need to do. Just give me a plan and I'll follow it.", 'Discuss the information and let me process it before committing to change.', 'Ask me what I think of the information. Help me to figure out what changes to make.', "Back off. I'll let you know what I'm interested in working on. I like to make these decisions."]
Journey	How much medical support or treatment do you need to get through the day?	['Not at all', 'A little', 'A moderate amount', 'Very much', 'An extreme amount']
Lifestyle	How often do you do MODERATE activities for at least 10 minutes? Moderate activities cause only light sweating or a moderate increase in breathing or heart rate (e.g., brisk walking, easy biking, tennis doubles, heavy cleaning or yard work).	['At least 7 times per week', 'At least 5 times a week', 'At least 3 times a week', '1-2 times per week', 'Less than once per week', 'Rarely or never']
Lifestyle	When you do MODERATE activities, for how long do you typically do these activities?	['N/A', '10 min', '20 min', '30 min', '45 min', '60 min', 'More than 60 min']
Lifestyle	How often do you do VIGOROUS activities for at least 10 minutes? Vigorous activities cause heavy sweating or large increases in breathing and heart rate (e.g., hiking, jogging at 6 mph, fast bicycling, playing soccer or basketball, tennis - singles).	['At least 7 times per week', 'At least 5 times a week', 'At least 3 times a week', '1-2 times per week', 'Less than once per week', 'Rarely or never']
Lifestyle	When you do VIGOROUS activities, for how long do you typically do these activities?	['N/A', '10 min', '20 min', '30 min', '45 min', '60 min', 'More than 60 min']
Lifestyle	On an average weekday, how much time do you spend seated?	['Most of the time (work plus relaxing, 12 hours)', 'Much of the time (work, 8 hours)', 'Sometimes (less than 8

©2017-2018 Arivale Inc. All Rights Reserved. Confidential and Proprietary.

Arivale
Your scientific path to wellness

		hours)', 'Almost never (less than one hour)']
Lifestyle	Do you walk or bike to work, school, or to do errands?	['Yes', 'No', 'Sometimes']
Lifestyle	Do you currently use tobacco?	['No', 'Rarely/Occasionally', 'Yes']
Lifestyle	My personal relationship status is	['Single', 'Partnered', 'Married', 'Divorced/Separated', 'Widowed']
Lifestyle	How many children do you have?	['None', 'One', 'Two', 'Three', 'Four or more']
Lifestyle	How many grandchildren do you have?	['None', 'One', 'Two', 'Three', 'Four or more']
Lifestyle	I share my home with:	['I live alone', 'Spouse/partner', 'Spouse/partner and Children', 'Friend or housemate', 'Children but no spouse/partner']
Lifestyle	Do you regularly spend time with friends?	['No', 'Yes']
Lifestyle	Do you wish you had more close friends?	['No', 'Yes']
Lifestyle	Do you currently have a membership to a gym or fitness/yoga studio?	['Yes', 'No']
Lifestyle	Do you use marijuana?	['No', 'Yes']
Lifestyle	Highest level of education received	['Less than high school', 'High school diploma or GED', 'Some college and/or technical school', "Associate and/or bachelor's degree", "Master's and/or doctorate and/or professional degree"]
Lifestyle	Did you use tobacco in the past?	['No', 'Rarely/Occasionally', 'Yes']
Lifestyle	For how many years have you been using tobacco?	['No', 'Rarely/Occasionally', 'Yes']
Lifestyle	For how many years did you use tobacco?	['No', 'Rarely/Occasionally', 'Yes']
Lifestyle	Are you exposed to second-hand smoke?	['No', 'Rarely/Occasionally', 'Yes']
Lifestyle	Do you use recreational drugs other than marijuana?	['No', 'Rarely/Occasionally', 'Yes']

©2017-2018 Arivale Inc. All Rights Reserved. Confidential and Proprietary.

Lifestyle	On an average night I sleep (round to the nearest hour)	['Less than 4 hours', '4 hours', '5 hours', '6 hours', '7 hours', '8 hours', '9 hours', 'More than 9 hours']
Lifestyle	On average, how many times do you wake up during the night?	['Not during the past month', 'Less than once a week', 'Once or twice a week', 'Three or more times a week']
Lifestyle	In the past six months, how would you rate your sleep?	['Poor', 'Fair', 'Average', 'Good', 'Excellent']
Lifestyle	In the past six months, how has lack of sleep affected your productivity, motivation, and ability to concentrate?	['Not at all', 'A little', 'A moderate amount', 'A lot', 'An extreme amount']
Lifestyle	I consider myself a morning person.	['Strongly disagree', 'Disagree', 'Neither disagree nor agree', 'Agree', 'Strongly agree']
Lifestyle	How often do you have trouble falling or staying asleep?	['Never or less than once a month', 'Less than one night per week', '1-2 nights per week', '3 nights or more per week']
Lifestyle	Do you have a pet?	['No', 'Yes']
Lifestyle	What types of MODERATE activities do you typically do?	['N/A', '10 min', '20 min', '30 min', '45 min', '60 min', 'More than 60 min']
Lifestyle	What types of VIGOROUS activities do you typically do?	['N/A', '10 min', '20 min', '30 min', '45 min', '60 min', 'More than 60 min']
Lifestyle	How often do you do MUSCLE STRENGTHENING activities (e.g. weight training, sit-ups, push-ups)?	['At least 7 times per week', 'At least 5 times a week', 'At least 3 times a week', '1-2 times per week', 'Less than once per week', 'Rarely or never']
Lifestyle	When you do MUSCLE STRENGTHENING activities, for how long do you typically do these activities?	['N/A', '10 min', '20 min', '30 min', '45 min', '60 min', 'More than 60 min']
Lifestyle	What types of MUSCLE STRENGTHENING activities do you typically do?	['N/A', '10 min', '20 min', '30 min', '45 min', '60 min', 'More than 60 min']
Lifestyle	How often do you do STRETCHING/FLEXIBILITY activities (e.g. yoga, balance)?	['At least 7 times per week', 'At least 5 times a week', 'At least 3 times a week', '1-2 times per

©2017-2018 Arivale Inc. All Rights Reserved. Confidential and Proprietary.

		week', 'Less than once per week', 'Rarely or never']
Lifestyle	When you do STRETCHING/FLEXIBILITY activities, for how long do you typically do these activities?	['N/A', '10 min', '20 min', '30 min', '45 min', '60 min', 'More than 60 min']
Lifestyle	What types of STRETCHING/FLEXIBILITY activities do you typically do?	['N/A', '10 min', '20 min', '30 min', '45 min', '60 min', 'More than 60 min']
Lifestyle	Do you have people in your life (other than family) that you trust and go to for support?	['Yes', 'No']
Lifestyle	What major type of tobacco did you use?	['Cigarettes', 'Cigar', 'Pipe', 'Vaping', 'Smokeless tobacco (e.g. chew or dip)']
Lifestyle	What major type of tobacco do you use?	['Cigarettes', 'Cigar', 'Pipe', 'Vaping', 'Smokeless tobacco (e.g. chew or dip)']
Lifestyle	How many packs a day?	['Less than half a pack/day', 'Half a pack/day', '1 pack/day', '1 and a half packs/day', '2 packs/day', 'More than 2 packs/day']
Lifestyle	How many packs a day?	['Less than half a pack/day', 'Half a pack/day', '1 pack/day', '1 and a half packs/day', '2 packs/day', 'More than 2 packs/day']
Lifestyle	For how many years have you been using marijuana?	['No', 'Rarely/Occasionally', 'Yes']
Lifestyle	What method do you most commonly use to smoke or consume marijuana?	['Inhalation (e.g. smoking or vaporizing)', 'Oral (e.g. edible)', 'Topical (e.g. oil)', 'Other']
Lifestyle	How often do you use marijuana?	['Less than once per month', '1-3 times per month', 'Once per week', '2-4 times per week', '5-6 times per week', 'Once per day', '2-3 times per day', '4-5 times per day', '6+ times per day']
Lifestyle	Did you use marijuana in the past?	['No', 'Rarely/Occasionally', 'Yes']

©2017-2018 Arivale Inc. All Rights Reserved. Confidential and Proprietary.

Lifestyle	For how many years did you use marijuana?	['No', 'Rarely/Occasionally', 'Yes']
Lifestyle	What method did you most commonly use to smoke or consume marijuana?	['Inhalation (e.g. smoking or vaporizing)', 'Oral (e.g. edible)', 'Topical (e.g. oil)', 'Other']
Lifestyle	How often did you use marijuana?	['Less than once per month', '1-3 times per month', 'Once per week', '2-4 times per week', '5-6 times per week', 'Once per day', '2-3 times per day', '4-5 times per day', '6+ times per day']
Personality	I am the life of the party.	['Strongly disagree', 'Disagree', 'Neither disagree nor agree', 'Agree', 'Strongly agree']
Personality	I feel little concern for others.	['Strongly agree', 'Agree', 'Neither disagree nor agree', 'Disagree', 'Strongly disagree']
Personality	I am always prepared.	['Strongly disagree', 'Disagree', 'Neither disagree nor agree', 'Agree', 'Strongly agree']
Personality	I get stressed out easily.	['Strongly agree', 'Agree', 'Neither disagree nor agree', 'Disagree', 'Strongly disagree']
Personality	I have a rich vocabulary.	['Strongly disagree', 'Disagree', 'Neither disagree nor agree', 'Agree', 'Strongly agree']
Personality	I don't talk a lot.	['Strongly agree', 'Agree', 'Neither disagree nor agree', 'Disagree', 'Strongly disagree']
Personality	I am interested in people.	['Strongly disagree', 'Disagree', 'Neither disagree nor agree', 'Agree', 'Strongly agree']
Personality	I leave my belongings around.	['Strongly agree', 'Agree', 'Neither disagree nor agree', 'Disagree', 'Strongly disagree']
Personality	I am relaxed most of the time.	['Strongly disagree', 'Disagree', 'Neither disagree nor agree', 'Agree', 'Strongly agree']
Personality	I have difficulty understanding abstract ideas.	['Strongly agree', 'Agree', 'Neither disagree nor agree', 'Disagree', 'Strongly disagree']

©2017-2018 Arivale Inc. All Rights Reserved. Confidential and Proprietary.

Personality	I feel comfortable around people.	['Strongly disagree', 'Disagree', 'Neither disagree nor agree', 'Agree', 'Strongly agree']
Personality	I insult people.	['Strongly agree', 'Agree', 'Neither disagree nor agree', 'Disagree', 'Strongly disagree']
Personality	I pay attention to details.	['Strongly disagree', 'Disagree', 'Neither disagree nor agree', 'Agree', 'Strongly agree']
Personality	I worry about things.	['Strongly agree', 'Agree', 'Neither disagree nor agree', 'Disagree', 'Strongly disagree']
Personality	I have a vivid imagination.	['Strongly disagree', 'Disagree', 'Neither disagree nor agree', 'Agree', 'Strongly agree']
Personality	I keep in the background.	['Strongly agree', 'Agree', 'Neither disagree nor agree', 'Disagree', 'Strongly disagree']
Personality	I sympathize with others' feelings.	['Strongly disagree', 'Disagree', 'Neither disagree nor agree', 'Agree', 'Strongly agree']
Personality	I make a mess of things.	['Strongly agree', 'Agree', 'Neither disagree nor agree', 'Disagree', 'Strongly disagree']
Personality	I seldom feel blue.	['Strongly disagree', 'Disagree', 'Neither disagree nor agree', 'Agree', 'Strongly agree']
Personality	I am not interested in abstract ideas.	['Strongly agree', 'Agree', 'Neither disagree nor agree', 'Disagree', 'Strongly disagree']
Personality	I start conversations.	['Strongly disagree', 'Disagree', 'Neither disagree nor agree', 'Agree', 'Strongly agree']
Personality	I am not interested in other people's problems.	['Strongly agree', 'Agree', 'Neither disagree nor agree', 'Disagree', 'Strongly disagree']
Personality	I get chores done right away.	['Strongly disagree', 'Disagree', 'Neither disagree nor agree', 'Agree', 'Strongly agree']
Personality	I am easily disturbed.	['Strongly agree', 'Agree', 'Neither disagree nor agree', 'Disagree', 'Strongly disagree']
Personality	I have excellent ideas.	['Strongly disagree', 'Disagree', 'Neither disagree nor agree', 'Agree', 'Strongly agree']

©2017-2018 Arivale Inc. All Rights Reserved. Confidential and Proprietary.

Personality	I have little to say.	['Strongly agree', 'Agree', 'Neither disagree nor agree', 'Disagree', 'Strongly disagree']
Personality	I have a soft heart.	['Strongly disagree', 'Disagree', 'Neither disagree nor agree', 'Agree', 'Strongly agree']
Personality	I often forget to put things back in their proper place.	['Strongly agree', 'Agree', 'Neither disagree nor agree', 'Disagree', 'Strongly disagree']
Personality	I get upset easily.	['Strongly agree', 'Agree', 'Neither disagree nor agree', 'Disagree', 'Strongly disagree']
Personality	I do not have a good imagination.	['Strongly agree', 'Agree', 'Neither disagree nor agree', 'Disagree', 'Strongly disagree']
Personality	I talk to a lot of different people at parties.	['Strongly disagree', 'Disagree', 'Neither disagree nor agree', 'Agree', 'Strongly agree']
Personality	I am not really interested in others.	['Strongly agree', 'Agree', 'Neither disagree nor agree', 'Disagree', 'Strongly disagree']
Personality	I like order.	['Strongly disagree', 'Disagree', 'Neither disagree nor agree', 'Agree', 'Strongly agree']
Personality	I change my mood a lot.	['Strongly agree', 'Agree', 'Neither disagree nor agree', 'Disagree', 'Strongly disagree']
Personality	I am quick to understand things.	['Strongly disagree', 'Disagree', 'Neither disagree nor agree', 'Agree', 'Strongly agree']
Personality	I don't like to draw attention to myself.	['Strongly agree', 'Agree', 'Neither disagree nor agree', 'Disagree', 'Strongly disagree']
Personality	I take time out for others.	['Strongly disagree', 'Disagree', 'Neither disagree nor agree', 'Agree', 'Strongly agree']
Personality	I shirk my duties.	['Strongly agree', 'Agree', 'Neither disagree nor agree', 'Disagree', 'Strongly disagree']
Personality	I have frequent mood swings.	['Strongly agree', 'Agree', 'Neither disagree nor agree', 'Disagree', 'Strongly disagree']
Personality	I use difficult words.	['Strongly disagree', 'Disagree', 'Neither disagree nor agree', 'Agree', 'Strongly agree']

©2017-2018 Arivale Inc. All Rights Reserved. Confidential and Proprietary.

Personality	I don't mind being the center of attention.	['Strongly disagree', 'Disagree', 'Neither disagree nor agree', 'Agree', 'Strongly agree']
Personality	I feel others' emotions.	['Strongly disagree', 'Disagree', 'Neither disagree nor agree', 'Agree', 'Strongly agree']
Personality	I follow a schedule.	['Strongly disagree', 'Disagree', 'Neither disagree nor agree', 'Agree', 'Strongly agree']
Personality	I get irritated easily.	['Strongly agree', 'Agree', 'Neither disagree nor agree', 'Disagree', 'Strongly disagree']
Personality	I spend time reflecting on things.	['Strongly disagree', 'Disagree', 'Neither disagree nor agree', 'Agree', 'Strongly agree']
Personality	I am quiet around strangers.	['Strongly agree', 'Agree', 'Neither disagree nor agree', 'Disagree', 'Strongly disagree']
Personality	I make people feel at ease.	['Strongly disagree', 'Disagree', 'Neither disagree nor agree', 'Agree', 'Strongly agree']
Personality	I am exacting in my work.	['Strongly disagree', 'Disagree', 'Neither disagree nor agree', 'Agree', 'Strongly agree']
Personality	I often feel blue.	['Strongly agree', 'Agree', 'Neither disagree nor agree', 'Disagree', 'Strongly disagree']
Personality	I am full of ideas.	['Strongly disagree', 'Disagree', 'Neither disagree nor agree', 'Agree', 'Strongly agree']
Stress	On a scale from 1-10 how would you rate your average stress level in the past month (1=no stress at all, 10=the most stress I've ever felt)	['1', '2', '3', '4', '5', '6', '7', '8', '9', '10']
Stress	Are you actively practicing a stress management technique?	['No', 'Yes']
Stress	In the last month, how often have you felt that you were unable to control the important things in your life?	['Never', 'Almost Never', 'Sometimes', 'Fairly Often', 'Very Often']
Stress	In the last month, how often have you felt confident about	['Very Often', 'Fairly Often', 'Sometimes', 'Almost Never', 'Never']

©2017-2018 Arivale Inc. All Rights Reserved. Confidential and Proprietary.

	your ability to handle your personal problems?	
Stress	In the last month, how often have you felt that things were going your way?	['Very Often', 'Fairly Often', 'Sometimes', 'Almost Never', 'Never']
Stress	In the last month, how often have you felt difficulties were piling up so high that you could not overcome them?	['Never', 'Almost Never', 'Sometimes', 'Fairly Often', 'Very Often']

Appendix E: Old Health History Assessment

The Arivale health history assessment was updated September 2017. The newer version is listed in Appendix D. The version below is the old version.

Assessment	Question Text	Options
Health History	Alcohol / Drug abuse	['Currently', 'Previously', 'Mother', 'Father', 'Siblings', 'Grandmother', 'Grandfather']
Health History	Allergy (Hay Fever)	['Currently', 'Previously', 'Mother', 'Father', 'Siblings', 'Grandmother', 'Grandfather']

©2017-2018 Arivale Inc. All Rights Reserved. Confidential and Proprietary.

Health History	Anemia	['Currently', 'Previously', 'Mother', 'Father', 'Siblings', 'Grandmother', 'Grandfather']
Health History	Arthritis (Rheumatoid)	['Currently', 'Previously', 'Mother', 'Father', 'Siblings', 'Grandmother', 'Grandfather']
Health History	Arthritis (Osteoarthritis)	['Currently', 'Previously', 'Mother', 'Father', 'Siblings', 'Grandmother', 'Grandfather']
Health History	Asthma	['Currently', 'Previously', 'Mother', 'Father', 'Siblings', 'Grandmother', 'Grandfather']
Health History	Bipolar Disorder	['Currently', 'Previously', 'Mother', 'Father', 'Siblings', 'Grandmother', 'Grandfather']
Health History	Bladder / Kidney Problems	['Currently', 'Previously', 'Mother', 'Father', 'Siblings', 'Grandmother', 'Grandfather']
Health History	Blood Clot (leg)	['Currently', 'Previously', 'Mother', 'Father', 'Siblings', 'Grandmother', 'Grandfather']
Health History	Blood Clot (lung)	['Currently', 'Previously', 'Mother', 'Father', 'Siblings', 'Grandmother', 'Grandfather']
Health History	Blood Transfusion	['Currently', 'Previously', 'Mother', 'Father', 'Siblings', 'Grandmother', 'Grandfather']
Health History	Breast Lump (benign)	['Currently', 'Previously', 'Mother', 'Father', 'Siblings', 'Grandmother', 'Grandfather']
Health History	Cancer Breast	['Currently', 'Previously', 'Mother', 'Father', 'Siblings', 'Grandmother', 'Grandfather']
Health History	Cancer Colon	['Currently', 'Previously', 'Mother', 'Father', 'Siblings', 'Grandmother', 'Grandfather']
Health History	Cancer Other Type	['Currently', 'Previously', 'Mother', 'Father', 'Siblings', 'Grandmother', 'Grandfather']
Health History	Cancer Ovarian	['Currently', 'Previously', 'Mother', 'Father', 'Siblings', 'Grandmother', 'Grandfather']
Health History	Cancer Prostate	['Currently', 'Previously', 'Mother', 'Father', 'Siblings', 'Grandmother', 'Grandfather']

©2017-2018 Arivale Inc. All Rights Reserved. Confidential and Proprietary.

Health History	Celiac Disease	['Currently', 'Previously', 'Mother', 'Father', 'Siblings', 'Grandmother', 'Grandfather']
Health History	Cataracts	['Currently', 'Previously', 'Mother', 'Father', 'Siblings', 'Grandmother', 'Grandfather']
Health History	Chicken Pox	['Currently', 'Previously', 'Mother', 'Father', 'Siblings', 'Grandmother', 'Grandfather']
Health History	Chronic Fatigue Syndrome	['Currently', 'Previously', 'Mother', 'Father', 'Siblings', 'Grandmother', 'Grandfather']
Health History	Colon Polyp	['Currently', 'Previously', 'Mother', 'Father', 'Siblings', 'Grandmother', 'Grandfather']
Health History	Coronary Artery Disease (including CAD, bypass surgery, and/or stent)	['Currently', 'Previously', 'Mother', 'Father', 'Siblings', 'Grandmother', 'Grandfather']
Health History	Concussion/TBI	['Currently', 'Previously', 'Mother', 'Father', 'Siblings', 'Grandmother', 'Grandfather']
Health History	Depression	['Currently', 'Previously', 'Mother', 'Father', 'Siblings', 'Grandmother', 'Grandfather']
Health History	Diabetes - Type 2	['Currently', 'Previously', 'Mother', 'Father', 'Siblings', 'Grandmother', 'Grandfather']
Health History	Diabetes - Type 1	['Currently', 'Previously', 'Mother', 'Father', 'Siblings', 'Grandmother', 'Grandfather']
Health History	Diverticulosis	['Currently', 'Previously', 'Mother', 'Father', 'Siblings', 'Grandmother', 'Grandfather']
Health History	Emphysema	['Currently', 'Previously', 'Mother', 'Father', 'Siblings', 'Grandmother', 'Grandfather']
Health History	Fractures (broken bones)	['Currently', 'Previously', 'Mother', 'Father', 'Siblings', 'Grandmother', 'Grandfather']
Health History	Fibromyalgia	['Currently', 'Previously', 'Mother', 'Father', 'Siblings', 'Grandmother', 'Grandfather']
Health History	Gallbladder Disease	['Currently', 'Previously', 'Mother', 'Father', 'Siblings', 'Grandmother', 'Grandfather']

©2017-2018 Arivale Inc. All Rights Reserved. Confidential and Proprietary.

Health History	Gastroesophageal Reflux (Heartburn/GERD)	['Currently', 'Previously', 'Mother', 'Father', 'Siblings', 'Grandmother', 'Grandfather']
Health History	Glaucoma	['Currently', 'Previously', 'Mother', 'Father', 'Siblings', 'Grandmother', 'Grandfather']
Health History	Gout	['Currently', 'Previously', 'Mother', 'Father', 'Siblings', 'Grandmother', 'Grandfather']
Health History	Graves Disease	['Currently', 'Previously', 'Mother', 'Father', 'Siblings', 'Grandmother', 'Grandfather']
Health History	Gynecological Conditions (Endometriosis)	['Currently', 'Previously', 'Mother', 'Father', 'Siblings', 'Grandmother', 'Grandfather']
Health History	Gynecological Conditions (Fibroids)	['Currently', 'Previously', 'Mother', 'Father', 'Siblings', 'Grandmother', 'Grandfather']
Health History	Gynecological Conditions (Other)	['Currently', 'Previously', 'Mother', 'Father', 'Siblings', 'Grandmother', 'Grandfather']
Health History	Heart Attack	['Currently', 'Previously', 'Mother', 'Father', 'Siblings', 'Grandmother', 'Grandfather']
Health History	Hepatitis Type A	['Currently', 'Previously', 'Mother', 'Father', 'Siblings', 'Grandmother', 'Grandfather']
Health History	Hepatitis Type B	['Currently', 'Previously', 'Mother', 'Father', 'Siblings', 'Grandmother', 'Grandfather']
Health History	Hepatitis Type C	['Currently', 'Previously', 'Mother', 'Father', 'Siblings', 'Grandmother', 'Grandfather']
Health History	Hepatitis Other	['Currently', 'Previously', 'Mother', 'Father', 'Siblings', 'Grandmother', 'Grandfather']
Health History	High Blood Pressure	['Currently', 'Previously', 'Mother', 'Father', 'Siblings', 'Grandmother', 'Grandfather']
Health History	High Cholesterol	['Currently', 'Previously', 'Mother', 'Father', 'Siblings', 'Grandmother', 'Grandfather']
Health History	Hip Fracture	['Currently', 'Previously', 'Mother', 'Father', 'Siblings', 'Grandmother', 'Grandfather']

©2017-2018 Arivale Inc. All Rights Reserved. Confidential and Proprietary.

Health History	Inflammatory Bowel Disease	['Currently', 'Previously', 'Mother', 'Father', 'Siblings', 'Grandmother', 'Grandfather']
Health History	Irritable Bowel Syndrome	['Currently', 'Previously', 'Mother', 'Father', 'Siblings', 'Grandmother', 'Grandfather']
Health History	Kidney Disease / Failure	['Currently', 'Previously', 'Mother', 'Father', 'Siblings', 'Grandmother', 'Grandfather']
Health History	Kidney Stones	['Currently', 'Previously', 'Mother', 'Father', 'Siblings', 'Grandmother', 'Grandfather']
Health History	Liver Disease	['Currently', 'Previously', 'Mother', 'Father', 'Siblings', 'Grandmother', 'Grandfather']
Health History	Lupus	['Currently', 'Previously', 'Mother', 'Father', 'Siblings', 'Grandmother', 'Grandfather']
Health History	Lyme Disease	['Currently', 'Previously', 'Mother', 'Father', 'Siblings', 'Grandmother', 'Grandfather']
Health History	Migraine Headaches	['Currently', 'Previously', 'Mother', 'Father', 'Siblings', 'Grandmother', 'Grandfather']
Health History	Multiple Sclerosis	['Currently', 'Previously', 'Mother', 'Father', 'Siblings', 'Grandmother', 'Grandfather']
Health History	Osteoporosis	['Currently', 'Previously', 'Mother', 'Father', 'Siblings', 'Grandmother', 'Grandfather']
Health History	Pneumonia	['Currently', 'Previously', 'Mother', 'Father', 'Siblings', 'Grandmother', 'Grandfather']
Health History	Prostate (enlargement)	['Currently', 'Previously', 'Mother', 'Father', 'Siblings', 'Grandmother', 'Grandfather']
Health History	Prostate (nodules)	['Currently', 'Previously', 'Mother', 'Father', 'Siblings', 'Grandmother', 'Grandfather']
Health History	Post Traumatic Stress Disorder	['Currently', 'Previously', 'Mother', 'Father', 'Siblings', 'Grandmother', 'Grandfather']
Health History	Seizure / Epilepsy	['Currently', 'Previously', 'Mother', 'Father', 'Siblings', 'Grandmother', 'Grandfather']

©2017-2018 Arivale Inc. All Rights Reserved. Confidential and Proprietary.

Health History	Skin Condition (Eczema)	['Currently', 'Previously', 'Mother', 'Father', 'Siblings', 'Grandmother', 'Grandfather']
Health History	Skin Condition (Psoriasis)	['Currently', 'Previously', 'Mother', 'Father', 'Siblings', 'Grandmother', 'Grandfather']
Health History	Skin Condition (Abnormal Moles)	['Currently', 'Previously', 'Mother', 'Father', 'Siblings', 'Grandmother', 'Grandfather']
Health History	Sleep Apnea	['Currently', 'Previously', 'Mother', 'Father', 'Siblings', 'Grandmother', 'Grandfather']
Health History	Stomach Ulcer	['Currently', 'Previously', 'Mother', 'Father', 'Siblings', 'Grandmother', 'Grandfather']
Health History	Stroke	['Currently', 'Previously', 'Mother', 'Father', 'Siblings', 'Grandmother', 'Grandfather']
Health History	Thyroid (Nodule)	['Currently', 'Previously', 'Mother', 'Father', 'Siblings', 'Grandmother', 'Grandfather']
Health History	Thyroid High (Overactive) / Hyperthyroidism	['Currently', 'Previously', 'Mother', 'Father', 'Siblings', 'Grandmother', 'Grandfather']
Health History	Thyroid Low (Underactive) / Hypothyroidism	['Currently', 'Previously', 'Mother', 'Father', 'Siblings', 'Grandmother', 'Grandfather']
Health History	Do you have any conditions not listed? Please list them here.	[None]
Health History	Diabetes - Gestational	['Currently', 'Previously', 'Mother', 'Father', 'Siblings', 'Grandmother', 'Grandfather']
Health History	Congestive Heart Failure	['Currently', 'Previously', 'Mother', 'Father', 'Siblings', 'Grandmother', 'Grandfather']
Health History	Peripheral Vascular Disease	['Currently', 'Previously', 'Mother', 'Father', 'Siblings', 'Grandmother', 'Grandfather']
Health History	Back pain or joint pain	['Currently', 'Previously', 'Mother', 'Father', 'Siblings', 'Grandmother', 'Grandfather']
Health History	Surgery in past 12 months	['Currently', 'Previously', 'Mother', 'Father', 'Siblings', 'Grandmother', 'Grandfather']

©2017-2018 Arivale Inc. All Rights Reserved. Confidential and Proprietary.

Function	KEGG Orthology (6,197), modules (409), pathways (146)

©2017-2018 Arivale Inc. All Rights Reserved. Confidential and Proprietary.
